# Genetic and environmental causes of variation in epigenetic aging across the lifespan

**DOI:** 10.1186/s13148-020-00950-1

**Published:** 2020-10-22

**Authors:** Shuai Li, Tuong L. Nguyen, Ee Ming Wong, Pierre-Antoine Dugué, Gillian S. Dite, Nicola J. Armstrong, Jeffrey M. Craig, Karen A. Mather, Perminder S. Sachdev, Richard Saffery, Joohon Sung, Qihua Tan, Anbupalam Thalamuthu, Roger L. Milne, Graham G. Giles, Melissa C. Southey, John L. Hopper

**Affiliations:** 1 Centre for Epidemiology and Biostatistics, Melbourne School of Population and Global Health, The University of Melbourne, Parkville, VIC 3051 Australia; 2 Centre for Cancer Genetic Epidemiology, Department of Public Health and Primary Care, University of Cambridge, Cambridge, UK; 3 Precision Medicine, School of Clinical Sciences At Monash Health, Monash University, Clayton, VIC Australia; 4 Department of Clinical Pathology, The University of Melbourne, Melbourne, VIC Australia; 5 Cancer Epidemiology Division, Cancer Council Victoria, Melbourne, Australia; 6 Mathematics and Statistics, Murdoch University, Perth, WA Australia; 7 Centre for Molecular and Medical Research, School of Medicine, Faculty of Health, Deakin University, Waurn Ponds, VIC Australia; 8 Centre for Healthy Brain Ageing (CHeBA), School of Psychiatry, University of New South Wales, Sydney, NSW Australia; 9 Neuropsychiatric Institute, Prince of Wales Hospital, Randwick, NSW Australia; 10 Murdoch Childrens Research Institute, Parkville, VIC Australia; 11 Institute of Health and Environment, Seoul National University, 1 Gwanak-ro, Gwanak-gu, Seoul, 151-742 Korea; 12 Epidemiology and Biostatistics, Department of Public Health, University of Southern Denmark, Odense, Denmark

**Keywords:** Aging, Epigenetic aging, Biological age, Epigenetic clock, DNA methylation, Twin study

## Abstract

**Background:**

DNA methylation-based biological age (DNAm age) is an important biomarker for adult health. Studies in specific age ranges have found widely varying results about its genetic and environmental causes of variation. However, these studies are not able to provide a comprehensive view of the causes of variation over the lifespan.

**Results:**

In order to investigate the genetic and environmental causes of DNAm age variation across the lifespan, we pooled genome-wide DNA methylation data for 4217 people aged 0–92 years from 1871 families. DNAm age was calculated using the Horvath epigenetic clock. We estimated familial correlations in DNAm age for monozygotic (MZ) twin, dizygotic (DZ) twin, sibling, parent–offspring, and spouse pairs by cohabitation status. Genetic and environmental variance components models were fitted and compared. We found that twin pair correlations were − 0.12 to 0.18 around birth, not different from zero (all *P* > 0.29). For all pairs of relatives, their correlations increased with time spent living together (all *P* < 0.02) at different rates (MZ > DZ and siblings > parent–offspring; *P* < 0.001) and decreased with time spent living apart (*P* = 0.02) at similar rates. These correlation patterns were best explained by cohabitation-dependent shared environmental factors, the effects of which were 1.41 (95% confidence interval [CI] 1.16 to 1.66) times greater for MZ pairs than for DZ and sibling pairs, and the latter were 2.03 (95% CI 1.13 to 9.47) times greater than for parent–offspring pairs. Genetic factors explained 13% (95% CI − 10 to 35%) of variation (*P* = 0.27). Similar results were found for another two epigenetic clocks, suggesting that our observations are robust to how DNAm age is measured. In addition, results for the other clocks were consistent with there also being a role for prenatal environmental factors in determining their variation.

**Conclusions:**

Variation in DNAm age is mostly caused by environmental factors, including those shared to different extents by relatives while living together and whose effects persist into old age. The equal environment assumption of the classic twin study might not hold for epigenetic aging.

## Background

Epigenetic alteration is considered to be a hallmark of aging [1]. Several measures of biological age based on DNA methylation (DNAm age) have been developed [2, 3] and found to be associated with mortality and disease risk in adulthood [2–6]. DNAm age, therefore, is potentially an important biomarker for adult health.

Lifestyle factors, disease risk factors, and genetic variants have been reported to be associated with DNAm age [2–4, 7–10]. Pedigree-based and single nucleotide polymorphism (SNP)-based studies have given widely varying estimates of the proportion of variation in DNAm age explained by genetic factors, ranging from 0 to 100% [6–9, 11–13]. One possible reason for this is that these studies focused on specific age ranges only. There is also evidence that environmental factors shared within families explain a substantial proportion of variation in the middle age [14]. Individual studies of specific age ranges are not able to provide a comprehensive view of the causes of variation over the lifespan.

We previously pooled DNA methylation data from a variety of twin and family studies in which participants were at different life stages, from birth to older age. We found evidence that variation in genome-wide average methylation is caused to a great extent by prenatal environmental factors, as well as by environmental factors shared by relatives (including spouse pairs) when they cohabit and that these effects can persist at least to some extent across the whole lifetime [15]. If specific age ranges were studied separately, these findings might not have been found.

We have now applied the same approach to investigate the genetic, shared environmental, and individual-specific environmental causes of variation in DNAm age across the lifespan.

## Results

### Sample characteristics

We analyzed genome-wide DNA methylation data from 10 studies (Additional file 1). The total sample included 4217 people aged 0–92 years from 1871 families, including monozygotic (MZ) twins, dizygotic (DZ) twins, siblings, parents, and spouses (Table 1).

**Table 1:** Sample characteristics by study

Study^a^	Biological sample	Microarray	Type of family members	*N* (females)	Chronological age, mean (SD)	DNAm age, mean (SD)	Absolute deviation of DNAm and chronological ages, mean (SD)	Epigenetic age acceleration, mean (SD)
PETS EPIC	Cord blood	EPIC	MZ	46 (24)	0 (0)	1.1 (0.4)	1.15 (0.38)	− 0.04 (0.38)
			DZ	44 (21)	0 (0)	1.2 (0.4)	1.22 (0.43)	0.04 (0.43)
PETS 27K CMBCs	Cord blood mononuclear cells (CMBCs)	27K	MZ	34 (18)	0 (0)	0.1 (0.3)	0.20 (0.16)	− 0.09 (0.25)
			DZ	18 (4)	0 (0)	0.3 (0.5)	0.35 (0.46)	0.16 (0.50)
PETS 27K HUVECs	Human umbilical vascular endothelial cells (HUVECs)	27K	MZ	26 (14)	0 (0)	6.2 (4.1)	6.15 (4.10)	− 0.95 (4.10)
			DZ	16 (4)	0 (0)	8.6 (5.1)	8.64 (5.07)	1.54 (5.07)
PETS 27K placenta	Placenta	27K	MZ	16 (12)	0 (0)	0.0 (0.2)	0.18 (0.10)	0.05 (0.21)
			DZ	12 (5)	0 (0)	− 0.1 (0.2)	0.21 (0.10)	− 0.07 (0.21)
PETS 450K birth	Buccal cells	450K	MZ	18 (8)	0 (0)	0.8 (0.4)	0.81 (0.37)	0.02 (0.37)
			DZ	10 (4)	0 (0)	0.8 (0.2)	0.75 (0.15)	− 0.04 (0.15)
PETS 450K 18 months	Buccal cells	450K	MZ	12 (6)	1.5 (0)	2.2 (0.7)	0.73 (0.66)	0.10 (0.66)
			DZ	8 (2)	1.5 (0)	2.0 (0.5)	0.49 (0.45)	− 0.15 (0.46)
BSGS	Peripheral blood	450K	MZ	134 (62)	13.8 (1.9)	18.4 (3.6)	4.65 (2.64)	− 0.34 (2.68)
			DZ	222 (107)	13.2 (2.0)	18.0 (3.7)	4.82 (2.66)	0.09 (2.63)
			Sibling	119 (59)	15.5 (2.8)	21.2 (4.8)	5.74 (2.96)	0.22 (2.84)
			Spouse/parents	139 (73)	46.6 (5.6)	50.8 (5.2)	4.75 (3.27)	0.00 (3.52)
E-Risk	Peripheral blood	450K	MZ	852 (414)	18 (0)	24.1 (3.7)	6.25 (3.40)	− 0.01 (3.67)
			DZ	612 (300)	18 (0)	24.1 (4.0)	6.38 (3.63)	0.03 (4.02)
DTR younger adults	Peripheral blood	450K	MZ	146 (66)	33.1 (2.0)	32.6 (4.8)	3.38 (2.71)	0.00 (4.31)
AMDTSS	Peripheral blood	450K	MZ	132 (132)	55.6 (8.4)	54.8 (6.7)	4.56 (3.51)	− 0.34 (4.58)
			DZ	132 (132)	57.0 (7.2)	56.5 (6.0)	5.04 (3.97)	0.60 (5.09)
			Sibling	215 (215)	56.6 (8.0)	55.5 (6.5)	5.04 (3.71)	− 0.16 (4.92)
TwinsUK	Peripheral blood	27K	MZ	66 (66)	58.4 (9.1)	56.0 (8.9)	4.23 (3.69)	− 0.75 (5.10)
			DZ	86 (86)	56.6 (7.7)	55.6 (8.1)	3.61 (2.80)	0.57 (4.14)
MuTHER	Adipose tissue	450K	MZ	186 (186)	61.0 (9.3)	58.9 (6.1)	4.78 (3.79)	− 0.19 (3.67)
			DZ	306 (306)	57.4 (9.3)	57.3 (6.2)	4.51 (3.25)	0.12 (3.57)
DTR older adults	Peripheral blood	450K	MZ	154 (78)	63.2 (4.1)	60.5 (6.7)	5.02 (3.77)	0.00 (5.63)
OATS	Peripheral blood	450K	MZ	216 (136)	71.2 (6.0)	65.5 (6.6)	6.67 (4.50)	0.00 (5.36)
LSADT 1997	Peripheral blood	450K	MZ	36 (22)	76.3 (2.0)	71.8 (3.7)	5.29 (3.12)	− 0.25 (3.86)
			DZ	50 (40)	76.2 (1.6)	72.2 (5.7)	5.62 (3.70)	0.18 (5.50)
LSADT 2007	Peripheral blood	450K	MZ	36 (22)	86.2 (2.0)	79.5 (4.9)	7.46 (4.56)	− 0.24 (4.85)
			DZ	50 (40)	86.1 (1.6)	80.0 (5.4)	7.13 (4.09)	0.18 (5.47)
MCCS	Peripheral blood	450K	Spouse	124 (62)	60.1 (6.2)	59.9 (8.1)	4.92 (3.88)	0.00 (6.19)

*EPIC* the HumanMethylationEPIC array, *27K* the HumanMethylation27 array, *450K* the HumanMethylation450 array, *MZ* monozygotic twin, *DZ* dizygotic twin, *N* sample size, *SD* standard deviation

^a^Studies—*PETS* Peri/postnatal Epigenetic Twins Study, including three datasets measured using the 27K array (using three biological samples), 450K array (at two points: at birth and age 18 months), and EPIC array, respectively; *BSGS* Brisbane System Genetics Study, *E-Risk* Environmental Risk Longitudinal Twin Study, *DTR* Danish Twin Registry, in two groups: younger and older adults, *AMDTSS* Australian Mammographic Density Twins and Sisters Study, *MuTHER* Multiple Tissue Human Expression Resource Study, *OATS* Older Australian Twins Study, *LSADT* Longitudinal Study of Aging Danish Twins, with samples collected at years 1997 and 2007, respectively, *MCCS* Melbourne Collaborative Cohort Study

DNAm age was calculated using the Horvath epigenetic clock [12] (https://dnamage.genetics.ucla.edu/new), as this clock is mostly applicable to our multi-tissue methylation data and study sample including newborns, children, and adults.

**Fig. 1:**
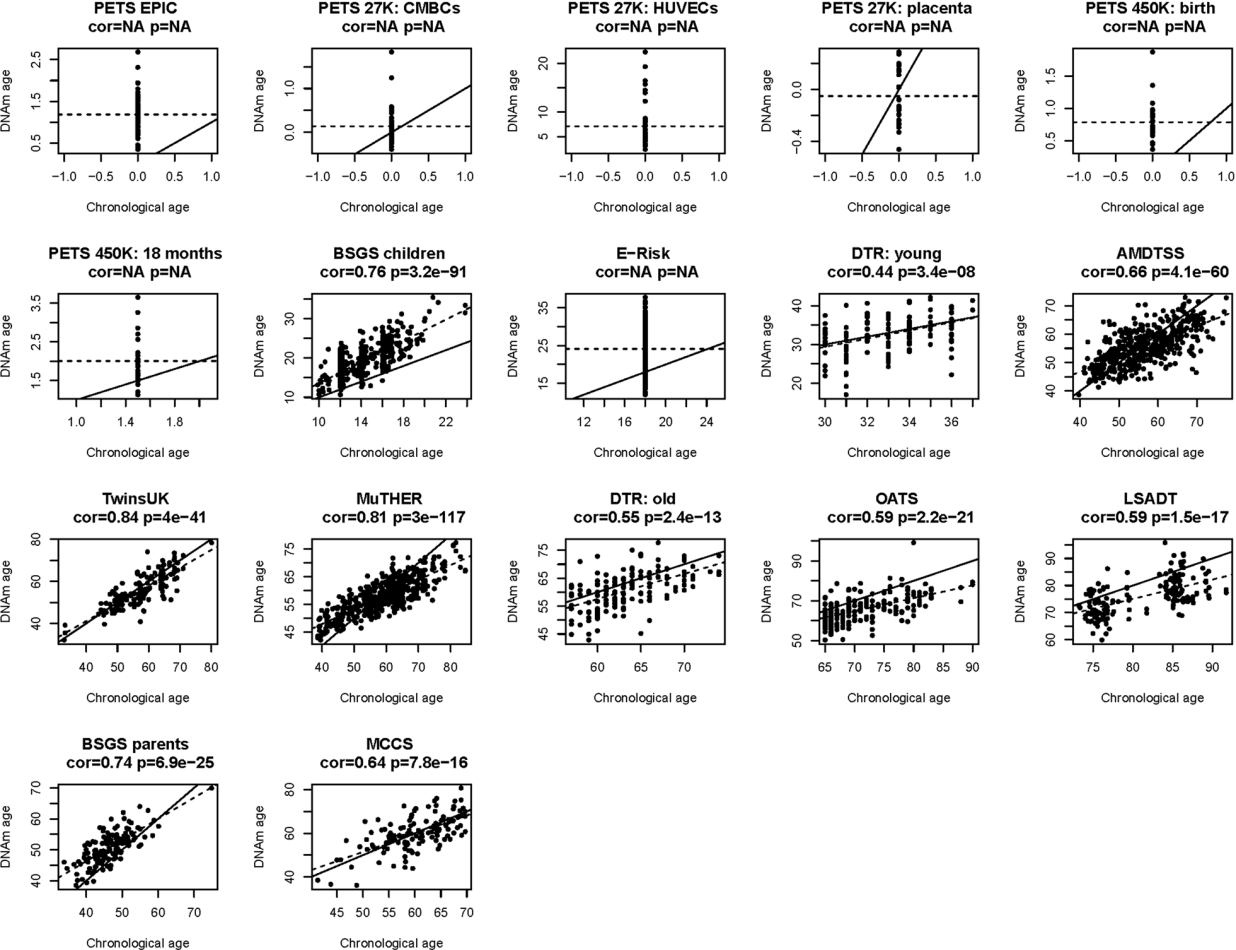
Correlation between chronological age and DNAm age measured by the epigenetic clock within each study. PETS: Peri/postnatal Epigenetic Twins Study, including three datasets measured using the 27K array, 450K array, and EPIC array, respectively; BSGS: Brisbane System Genetics Study; E-Risk: Environmental Risk Longitudinal Twin Study; DTR: Danish Twin Registry; AMDTSS: Australian Mammographic Density Twins and Sisters Study; MuTHER: Multiple Tissue Human Expression Resource Study; OATS: Older Australian Twins Study; LSADT: Longitudinal Study of Aging Danish Twins; MCCS: Melbourne Collaborative Cohort Study

**Fig. 2:**
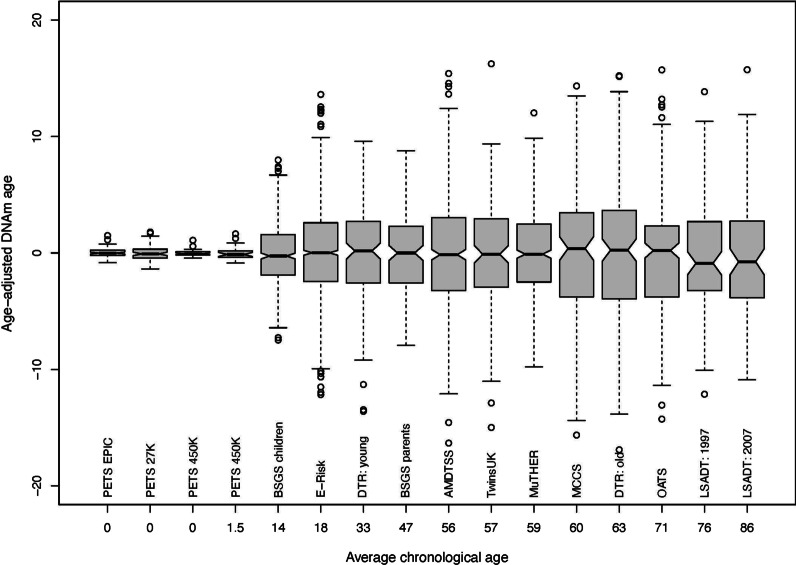
Variance in age-adjusted DNAm age measured by the epigenetic clock by chronological age. PETS: Peri/postnatal Epigenetic Twins Study, including three datasets measured using the 27K array, 450K array, and EPIC array, respectively; BSGS: Brisbane System Genetics Study; E-Risk: Environmental Risk Longitudinal Twin Study; DTR: Danish Twin Registry; AMDTSS: Australian Mammographic Density Twins and Sisters Study; MuTHER: Multiple Tissue Human Expression Resource Study; OATS: Older Australian Twins Study; LSADT: Longitudinal Study of Aging Danish Twins; MCCS: Melbourne Collaborative Cohort Study

DNAm age was moderately to strongly correlated with chronological age within each dataset, with correlations ranging from 0.44 to 0.84 (Fig. 1). The variance of DNAm age increased with chronological age, being small for newborns, greater for adolescents, and relatively constant with age for adults (Fig. 2). A similar pattern was observed for the absolute deviation between DNAm age and chronological age (Table 1). Within each study, MZ and DZ pairs had similar absolute deviations and residuals in DNAm age adjusted for chronological age.

### Within-study familial correlations

Table 2 shows the within-study familial correlation estimates. There was no difference in the correlation between MZ and DZ pairs for newborns or adults, but there was a difference (*P* < 0.001) for adolescents: 0.69 (95% confidence interval [CI] 0.63 to 0.74) for MZ pairs and 0.35 (95% CI 0.20 to 0.48) for DZ pairs. For MZ and DZ pairs combined, there was consistent evidence across datasets and tissues that the correlation was around − 0.12 to 0.18 at birth and 18 months, not different from zero (all *P* > 0.29), and about 0.3 to 0.5 for adults (different from zero in seven of eight datasets; all *P* < 0.01). Across all datasets, the results suggested that twin pair correlations increased with age from birth up until adulthood and were maintained to older age.

**Table 2:** Within-study familial correlations in DNAm age

Study^a^	Type of pairs	Number of pairs	Mean age	Correlation (95% CI)	*P*	*P* for MZ versus DZ
PETS EPIC	MZ	23	0	0.18 (− 0.28 to 0.57)	0.45	0.57
	DZ	22	0	0.01 (− 0.35 to 0.36)	0.98	
	MZ and DZ	45	0	0.15 (− 0.22 to 0.34)	0.64	
PETS 27K	MZ	22	0	0.35 (− 0.04 to 0.64)	0.85	0.69
	DZ	11	0	0.00 (− 0.46 to 0.46)	0.09	
	MZ and DZ	33	0	0.18 (− 0.15 to 0.47)	0.76	
PETS 450K birth	MZ	9	0	− 0.05 (− 0.51 to 0.43)	0.09	0.29
	DZ	5	0	0.62 (0.01 to 0.89)	0.99	
	MZ and DZ	14	0	− 0.08 (− 0.54 to 0.41)	0.29	
PETS 450K 18 months	MZ	6	1.5	− 0.06 (− 0.62 to 0.55)	0.87	0.58
	DZ	4	1.5	− 0.52 (− 0.91 to 0.40)	0.31	
	MZ and DZ	10	1.5	− 0.12 (− 0.62 to 0.46)	0.71	
BSGS	MZ	67	13.8	0.69 (0.63 to 0.74)	< 0.001	< 0.001
	DZ	111	13.2	0.35 (0.20 to 0.48)	< 0.001	
	Siblings	260	14.0	0.32 (0.20 to 0.42)	< 0.001	
	Parent–offspring	363	13.4	0.15 (0.02 to 0.27)	0.02	
	Spouses	59	46.6	− 0.01 (− 0.25 to 0.24)	0.96	
E-Risk	MZ	426	18.0	0.46 (0.40 to 0.52)	< 0.001	0.28
	DZ	306	18.0	0.40 (0.33 to 0.47)	< 0.001	
	MZ and DZ	732	18.0	0.43 (0.39 to 0.48)	< 0.001	
DTR younger adults	MZ	73	33.1	0.62 (0.52 to 0.70)	< 0.001	−
AMDTSS	MZ	66	55.6	0.43 (0.26 to 0.58)	< 0.001	0.20
	DZ	66	57.0	0.24 (0.04 to 0.42)	0.02	
	MZ and DZ	132	56.3	0.32 (0.18 to 0.45)	< 0.001	
	Siblings	552	56.4	0.12 (0.02 to 0.22)	0.02	
TwinsUK	MZ	33	55.3	0.23 (− 0.03 to 0.46)	0.09	0.41
	DZ	43	59.2	0.04 (− 0.33 to 0.39)	0.85	
	MZ and DZ	76	57.5	0.12 (− 0.07 to 0.37)	0.17	
MuTHER	MZ	93	58.4	0.54 (0.44 to 0.63)	< 0.001	0.08
	DZ	153	56.6	0.37 (0.25 to 0.48)	< 0.001	
	MZ and DZ	246	57.3	0.44 (0.36 to 0.52)	< 0.001	
DTR older adults	MZ	77	63.2	0.55 (0.43 to 0.65)	< 0.001	−
OATS	MZ	108	71.2	0.40 (0.26 to 0.53)	< 0.001	−
LSADT 1997	MZ	18	76.3	0.04 (− 0.66 to 0.70)	0.93	0.36
	DZ	25	76.2	0.39 (0.14 to 0.60)	< 0.001	
	MZ and DZ	43	76.2	0.34 (0.09 to 0.55)	0.01	
LSADT 2007	MZ	18	86.2	0.41 (0.05 to 0.68)	0.04	0.95
	DZ	25	86.1	0.40 (0.11 to 0.62)	0.01	
	MZ and DZ	43	86.1	0.40 (0.17 to 0.59)	< 0.001	
MCCS	Spouses	62	60.1	0.12 (− 0.12 to 0.35)	0.33	−

*MZ* monozygotic twin, *DZ* dizygotic twin, *CI* confidence interval

^a^Studies—*PETS* Peri/postnatal Epigenetic Twins Study, including three datasets measured using the 27K array, 450K array (at two points: at birth and age 18 months), and EPIC array, respectively; *BSGS* Brisbane System Genetics Study; *E-Risk* Environmental Risk Longitudinal Twin Study, *DTR* Danish Twin Registry, in two groups: younger and older adults, *AMDTSS* Australian Mammographic Density Twins and Sisters Study, *MuTHER* Multiple Tissue Human Expression Resource Study, *OATS* Older Australian Twins Study, *LSADT* Longitudinal Study of Aging Danish Twins, with samples collected at years 1997 and 2007, respectively, *MCCS* Melbourne Collaborative Cohort Study

The correlation for adolescent sibling pairs was 0.32 (95% CI 0.20 to 0.42), not different from that for adolescent DZ pairs (*P* = 0.89), but less than that for adolescent MZ pairs (*P* < 0.001). Middle-aged sibling pairs were correlated at 0.12 (95% CI 0.02 to 0.22), less than that for adolescent sibling pairs (*P* = 0.02). Parent–offspring pairs were correlated at 0.15 (95% CI 0.02 to 0.27), less than that for pairs of other types of first-degree relatives in the same study, e.g., DZ pairs and sibling pairs (both *P* < 0.04). The spouse-pair correlations were − 0.01 (95% CI − 0.25 to 0.24) and 0.12 (95% CI − 0.12 to 0.35).

From the sensitivity analysis, the familial correlation results were robust to the adjustment for blood cell composition (Additional file 1: Table S1).

### Familial correlations across the lifespan

From modeling the familial correlations for the different types of pairs as a function of their cohabitation status (Additional file 1: Table S2), the estimates of *θ* (see “Methods” section for definition) ranged from 0.76 to 1.20 across pairs, none different from 1 (all *P* > 0.1). We therefore fitted a model with *θ* = 1 for all pairs; the fit was not different from the model above (*P* = 0.69). Under the latter model, the familial correlations increased with time living together at different rates (*P* < 0.001) across pairs. The decreasing rates did not differ across pairs (*P* = 0.27). The correlations for DZ and sibling pairs were similar (*P* = 0.13), and when combined their correlation was different from that for parent–sibling pairs (*P* = 0.002) even though these pairs are all genetically first-degree relatives, and was smaller than that for the MZ pairs (*P* = 0.001).

**Fig. 3:**
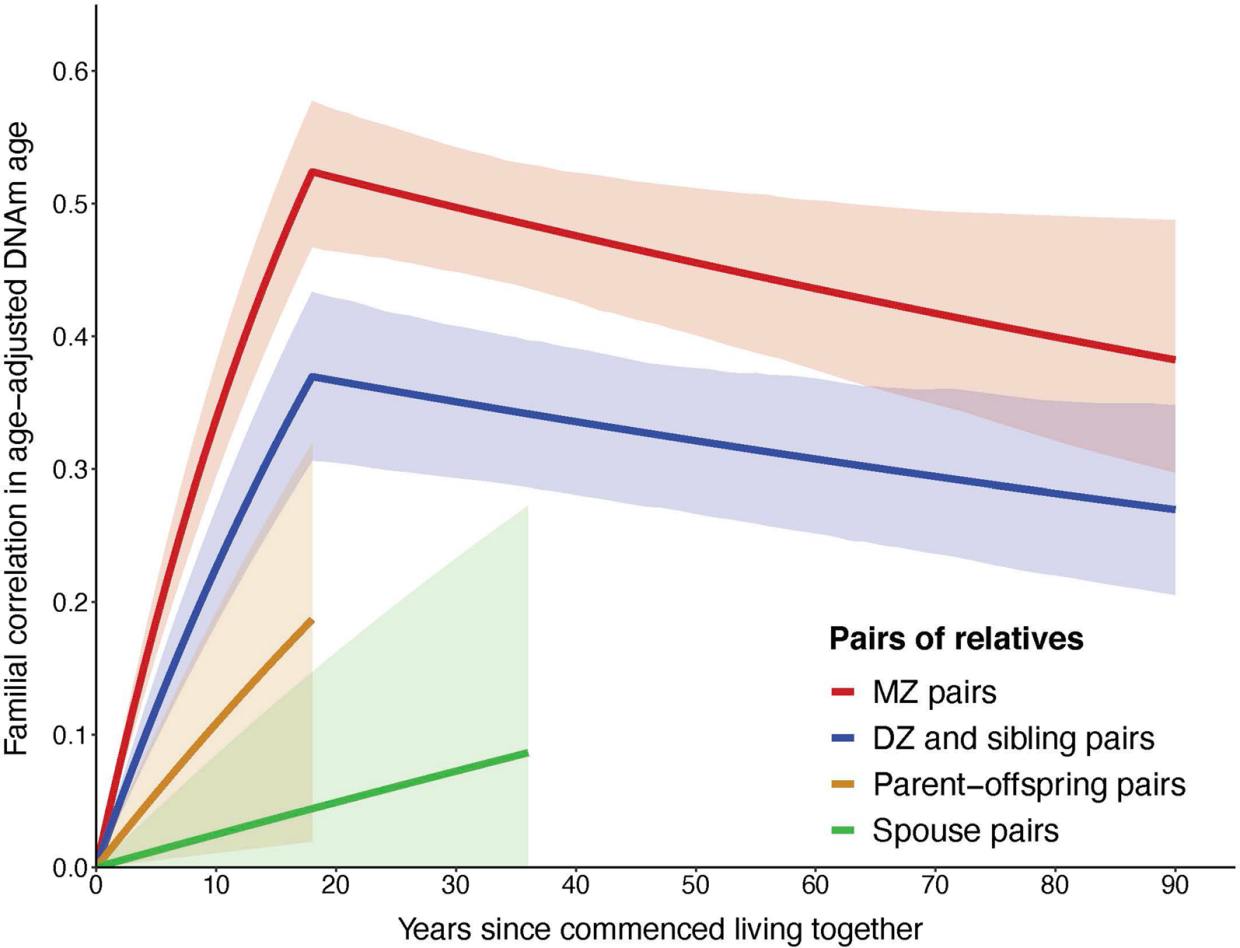
Familial correlations in DNAm age measured by the epigenetic clock for the different types of pairs across the lifespan. Lines are the predicted familial correlations from modeling the familial correlation as a function of cohabitation status, and shadows are the corresponding 95% confidence intervals. *MZ* monozygotic twin, *DZ* dizygotic twin

We then fitted a model in which DZ and sibling pairs were combined and the decreasing rates were the same across all pairs. The goodness of fit of this model was not inferior to that of the model above (*P* = 0.14), and the model included fewer parameters. Under this model, the familial correlations for MZ, DZ and sibling, and parent–offspring pairs all increased with time living together (all *P* < 0.02) with different increasing rates (*P* < 0.001); most rapidly for MZ pairs (*λ* = 0.041, 95% CI 0.035 to 0.048), less rapidly for DZ and sibling pairs (*λ* = 0.026, 95% CI 0.020 to 0.031), and least rapidly for parent–offspring pairs (*λ* = 0.011, 95% CI 0.002 to 0.0021), and decreased with time living apart (*P* = 0.02); see Fig. 3.

### Causes of variation across the lifespan

**Fig. 4:**
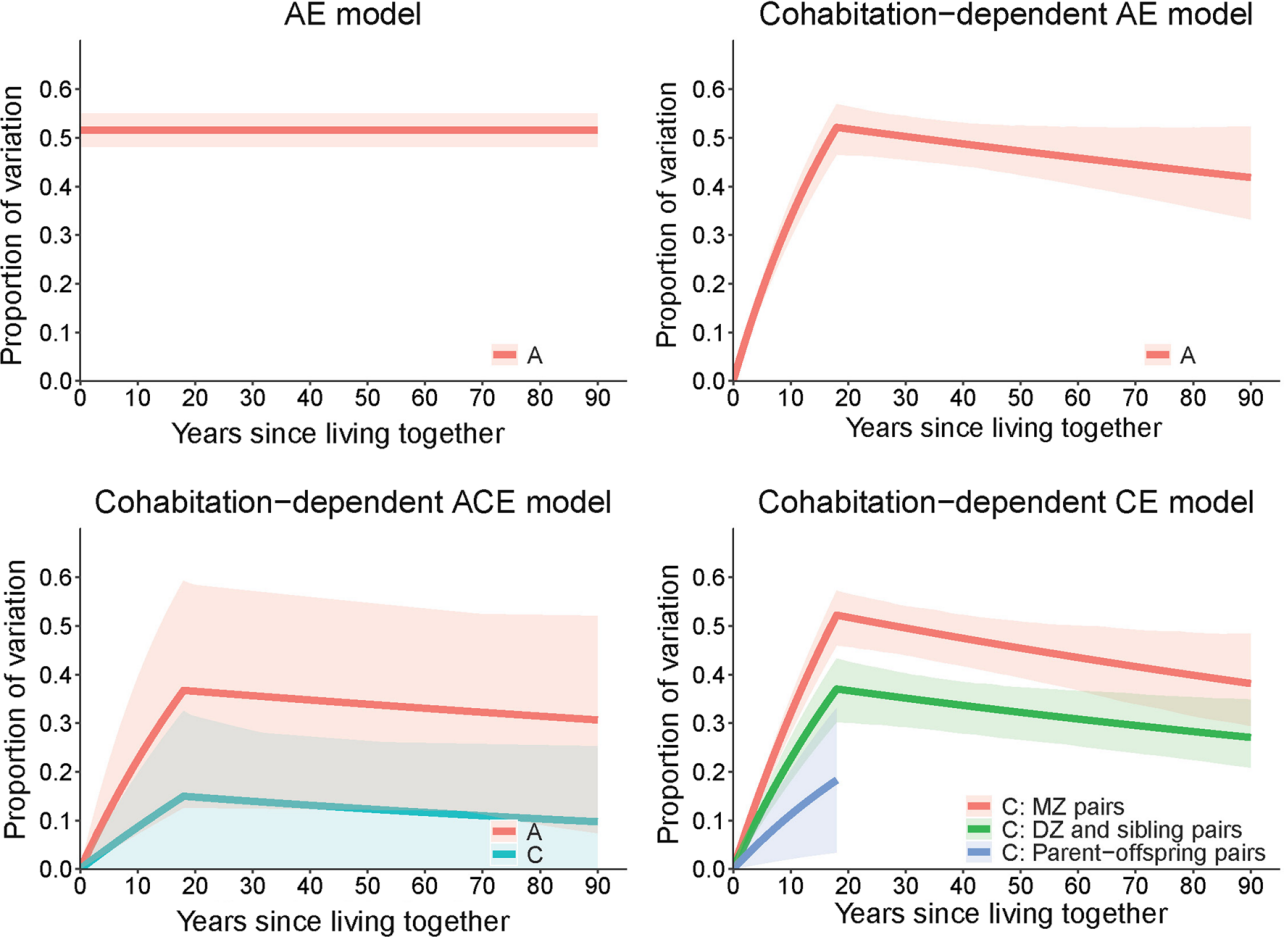
Proportion of variation in DNAm age measured by the epigenetic clock across the lifespan explained by genetic and environmental factors. Lines are the predicted proportions of variation explained by genetic and environmental factors from the variance components modeling, and shadows are the corresponding 95% confidence intervals. A: additive genetic factors; C: shared environmental factors; E: individual-specific environmental factors; *MZ* monozygotic twin, *DZ* Dizygotic twin. Model details—AE model: variation was assumed to be caused by only A and E, and the effects of A are constant across the lifespan; cohabitation-dependent AE model: variation was assumed to be caused by only A and E, and the effects of A depend on cohabitation; cohabitation-dependent ACE model: variation was assumed to be caused by A, C and E, and the effects of A and C both depend on cohabitation; cohabitation-dependent CE model: variation was assumed to be caused by only C and E, and the effects of C depend on cohabitation

Results from modeling the causes of variation across the lifespan are shown in Fig. 4 and Additional file 1: Table S3. Under the AE model, additive genetic factors explained 52% (95% CI 48 to 53%) of variation. This, however, was the worst fitting model. Under the cohabitation-dependent AE model, the effects of genetic factors increased with time living together and decreased with time living apart, and explained minimal variation around birth, ~ 40% of variation in adolescence and adulthood, and ~ 50% of variation at age of 18 years. Under the cohabitation-dependent ACE model, both the effects of genetic factors and the effects of shared environmental factors increased with time living together but did not change with time living apart. The goodness of fits of the cohabitation-dependent AE and cohabitation-dependent ACE models were similar.

The best-fitting model was the cohabitation-dependent CE model. Under this model, different pairs shared the effects of environmental factors to different extents. The effects for MZ pairs were 1.41 (95% CI 1.16 to 1.66) times those for DZ and sibling pairs, and the latter were 2.03 (95% CI 1.13 to 9.47) times those for parent–offspring pairs. For all pairs, the proportion of variation explained by shared environmental factors increased with time living together (*P* < 0.001) and decreased at a slower rate with time living apart (*P* = 0.02).

Under the above cohabitation-dependent CE model, we further assumed that the variation is additionally caused by genetic factors whose effects are constant across the lifespan. Genetic factors were estimated to explain 13% (95% CI − 10 to 35%) of the variation (*P* = 0.27). That is, after taking into account the existence of non-genetic cohabitation-dependent effects, there was no evidence for a substantive role of genetic factors.

### Results for other DNAm age measures

We also similarly studied two other DNAm age measures, a skin and blood clock developed by Horvath et al. [16] and a blood clock developed by Han et al. [17], which are also developed across tissues and/or ages. Overall, DNAm ages predicted by the two measures appeared to be more similar to chronological age than the DNAm age predicted by the Horvath epigenetic clock: within the same study, they had higher correlations with chronological age (Additional file 2: Figure S1, Additional file 3: Figure S2) and lower absolute deviations from chronological age (Additional file 1: Table S4). For both measures, MZ and DZ pairs had similar absolute deviations and residuals in DNAm age adjusted for chronological age. Similar to the DNAm age predicted by the Horvath epigenetic clock, the variance of the DNAm ages predicted by the two measures increased with age in early life and remained relatively constant with age in adulthood (Additional file 4: Figure S3, Additional file 5: Figure S4).

Additional file 1: Table S5 shows the within-study familial correlation results for the two measures. For both measures, similar results to those for the Horvath epigenetic clock were observed: twin pair correlations increased with age from birth to adulthood and decreased with age in adulthood; no evidence that the twin-pair correlations differed by zygosity was observed across the lifespan, except in adolescence and at age 18 years. For both measures, newborn twins were found to be correlated at ~ 0.4, different from zero (both *P* < 0.002) but not different by zygosity (both *P* > 0.1).

## Discussion

Our study provides novel insights into the causes of variation in DNAm age across the lifespan, which appear to be almost entirely environmental (i.e. non-genetic) factors. These include cohabitation-related environmental factors that are evident prior to adulthood, and whose effects persist across the whole of the lifespan. Two longitudinal studies have also found that DNAm age is largely set before adulthood [18].

Our data suggest that people in the same family are not correlated in DNAm age when they start cohabiting; the longer they live together, the more similar they become but at a rate that differs substantially depending on their relationship. This is likely due to the different types of relatives sharing environmental factors relevant to DNAm age to different degrees. When pairs of relatives live apart, they no longer share the cohabitation environment, and this is reflected by a slow dissipation of the effects of shared environmental factors across adulthood at a rate that appears to be similar for all pairs.

Our study is the first to provide a comprehensive view of the genetic and environmental causes of DNAm age variation across the lifespan. Focusing on limited age ranges or types of relatives might bias the interpretation for the causes. For example, if middle-aged (e.g., 40–70 years old) twins only (i.e., no siblings, parents or spouses) were studied, the higher MZ pair correlation compared with DZ pair correlation at that age range (see Fig. 3) might have been interpreted as being caused by genetic factors to some extent, as there are no data from other age ranges or types of relatives contributing to the interpretation. Without using data of various types of relatives whose ages cover the whole lifespan, the comprehensive view would have not been easily obtained.

For MZ pairs, some DNA methylation measures have been found to be similar at birth but divergent over the lifetime, a phenomenon called ‘epigenetic drift’ [15, 19]. DNAm age, however, shows a different pattern; MZ pairs are not similar at birth (and neither are DZ pairs) but become more similar the longer they live together, and do so more rapidly than do DZ or other pairs of relatives. In adulthood, MZ pairs then appear to slowly become less similar in DNAm age the longer they live apart, at the same rate as for other pairs of relatives, but still maintain a substantial similarity even into late life. These observations suggest that DNAm age reflects biological aging processes beyond what is reflected by DNA methylation alone.

Our finding that environmental factors shared while cohabiting play a major role in determining the variation in DNAm age is also supported by the observation that the variance of DNAm age increased dramatically with age prior to adulthood and was relatively stable across adulthood (Fig. 2, Additional file 4: Fgiure S3, Additional file 5: Figure S4). The latter has also been found by previous studies [18].

We investigated DNAm age based on other two pan-tissue/age clocks and found similar results to those for the Horvath clock. These results imply the role of cohabitation-related environmental factors in influencing the variation in these two clocks as well and suggest that our findings are robust to the way DNAm age is measured. These results of newborn MZ and DZ pairs were not differentially correlated in the two clocks implying the additional role of prenatal environmental factors in influencing the variation in these clocks, similar to what we found for the genome-wide average DNA methylation [15].

Given DNAm age has been found to be associated with the risks of death and various diseases in adulthood, identifying the environmental factors affecting DNAm age prior to adulthood might give novel insights into which, and how, early-life factors impact late-life health outcomes. This would have obvious implications for prevention and its timing. There is some evidence that DNAm age is associated with physical developmental characteristics, and exposures to stress and violence for children, although most studies had a moderate sample size [20–24].

The classic twin design assumes that MZ and DZ pairs share environmental effects relevant to the trait of interest to exactly the same extent, i.e., the equal environment assumption. Our study shows that this assumption might not hold for DNAm age because there was strong evidence that MZ and DZ pairs share their pre-adult environmental effects to different extents. Furthermore, DZ and sibling pairs were more correlated than parent–offspring pairs, despite all being genetically first-degree relatives of one another; this is not consistent with the correlations predicted by additive genetic factors. Given there is no substantive evidence of genetic effects, our results are not consistent with gene–environment interaction either [25]; we found that models including genetic effects, no matter whether as constant or cohabitation-dependent, were less consistent with the data compared with the cohabitation-CE model.

Previous twin and pedigree studies assumed the equal environment assumption holds perfectly and consequently reported the heritability of DNAm age to be ~ 40% in adolescence and middle age [6, 9, 12]. Note that under our cohabitation-dependent AE model (which makes the equal environment assumption), genetic factors would explain ~ 40% of variation in adolescence and middle age. This model, however, was not a good fit and was rejected in favor of models that included cohabitation-dependent environmental effects.

Studies have predicted that measured SNPs could explain 0–70% of variation in DNAm age measured from whole blood and brain tissue [7–9, 11]. Those analyses explicitly assumed, however, that all of the phenotypic covariance is due to genetic factors. In particular, one study predicted the SNP-based heritability of DNAm age based on mothers and children increased with the children’s age, being zero when the children were around birth and 37% when the children were 15 years old [7]—in line with our data and the estimates under the cohabitation-dependent AE model that was rejected. Without relying on the equal environment assumption, we found that genetic factors explained at most a small, and not statistically significant, proportion (~ 10%) of variation. Therefore, studies using the equal environment assumption might have overestimated the influence of genetic factors on DNAm age variation.

Our study has several strengths. One strength is that we have included participants whose ages covered the whole lifespan, so we could provide insights into the genetic and environmental causes of DNAm age variation which are unable to be provided by studies focusing on specific ages only. The other strength is that we have substantial sample size, even within studies, so we can detect moderate correlations with good precision, and have the power to distinguish between different variance components models. Our findings should be interpreted with caution, given that they are from statistical modeling which alone cannot prove that a consistent model is a true representation of nature. All that can be said is whether or not the data ‘are consistent with’ a particular explanation. Nonetheless, statistical modeling is an attempt to identify the plausible and implausible explanations of data, and our results suggest that cohabitation environmental factors being shared by pairs of relatives to different extents are more plausible than genetic explanations.

## Conclusions

The variation in epigenetic aging across the lifespan is most consistent with having been caused, at least to a large extent, by environmental factors, including those shared to different extents by relatives while living together. The effects of the cohabitation environment increase with the time living together and persist into old age. The equal environment assumption of the classic twin study might not hold for epigenetic aging. Given the relationships between DNAm age and health outcomes, these findings highlight the importance and potential of pre-adulthood prevention related to environmental factors for adult diseases and biological aging.

## Methods

### Study sample

We analyzed genome-wide DNA methylation data from 10 studies, most of which were accessed through public repositories: Peri/postnatal Epigenetic Twins Study (PETS), Brisbane System Genetics Study (BSGS), Environmental Risk Longitudinal Twin Study (E-Risk), Danish Twin Registry (DTR), Australian Mammographic Density Twins and Sisters Study (AMDTSS), TwinsUK cohort, Multiple Tissue Human Expression Resource (MuTHER) Study, Older Australian Twins Study (OATS), Longitudinal Study of Aging Danish Twins (LSADT), and Melbourne Collaborative Cohort Study (MCCS). The total sample included 4217 people aged 0–92 years from 1871 families. Most studies measured methylation using DNA extracted from peripheral blood and the HumanMethylation450 array (Table 1 and Additional file 1).

### Data preprocessing

As several datasets on public repositories contained quality-controlled and preprocessed data only, we were unable to apply the same preprocessing methods across datasets. We used the study-specific data preprocessing methods to address study-specific technical variations. This design allows us to investigate true biological signals independent of any bias introduced from a unifying data preprocessing approach. In DNAm age calculation, we chose the ‘Normalize Data’ option of the online calculator to normalize each dataset to be comparable to the training data of this epigenetic clock.

### DNAm age and epigenetic age acceleration

We used the Horvath epigenetic clock [12] to determine DNAm age (https://dnamage.genetics.ucla.edu/new) because it was developed across tissues and ages, and the 353 methylation sites used by this clock are common to the three methylation arrays used by the 10 studies (Table 1).

To adjust for the effects of chronological age on DNAm age, we studied epigenetic age acceleration, calculated as the residuals from a linear regression of DNAm age on chronological age. This calculation was done for each longitudinal measurement of the PETS 450K dataset and of the LSADT, for each generation of the BSGS, and for each age group of the DTR. For the PETS 27K dataset, DNAm age was standardized to have zero mean and unit variance for each type of biological sample, and the average standardized DNAm age across biological samples was used to calculate epigenetic age acceleration.

Sensitivity analyses were performed using only those studies in which DNA methylation was measured in blood to examine the robustness of results to cell composition. Naive CD8+ T cells, exhausted CD8+ T cells, plasmablasts, CD4+ T cells, natural killer cells, monocytes, and granulocytes estimated from the DNA methylation data [12, 26] were additionally adjusted for in calculating epigenetic age acceleration.

We studied two other DNAm age measures which were developed across tissues and/or ages too, so they might be also applicable to our data. One is the skin and blood clock developed using multi-tissue methylation data of a sample aged 0–94 years [16]. As some of the 391 methylation sites used by this clock were not included the PETS 450K and 27K datasets, these datasets were not included in its analysis. The other measure is developed by Han et al. [17] using a sample aged 1–101 years. As the measure is developed using HM450K array blood methylation data, non-blood or 27K datasets were not included in its analysis.

### Statistical analysis

Residuals of epigenetic age acceleration adjusted for sex were used in subsequent analyses. We used a multivariate normal model for pedigree analysis [27, 28] and the program FISHER [29] to estimate correlations for different types of pairs (MZ, DZ, sibling, parent–offspring and spouse) and to fit variance components models. The likelihood ratio test was used to compare nested models. All *P* values were two-sided, and *P* < 0.05 was considered significant.

According to the pattern in familial correlations by chronological age, and following previous theoretical and empirical studies [15, 27, 30], the familial correlations across the lifespan were modeled as a function of the cohabitation status of the pair. The modeling was performed using the pooled data across all studies. Study-specific variance in the residuals was used in analysis. For individuals *i* and *j* from the same family, their correlation was modeled as

$${\rho }_{ij}=\left\{\begin{array}{ll}\theta - {\text{e}}^{-\lambda t}& \quad\mathrm{if }\quad t\le {t}_{0}\\ (\theta - {\text{e}}^{-\lambda {t}_{0}}){\text{e}}^{-\upnu (t-{t}_{0})},&\quad \mathrm{if}\quad t>{t}_{0}\end{array}\right.$$

where 0 ≤ *θ* ≤ 2, and *λ*, *υ* ≥ 0.

Under this model, the correlation when the pairs start to live together is *θ* minus 1, and *λ* and *υ* reflect the increasing and decreasing rates at which the familial correlation increases with the length of cohabitation and decreases with the length of separation, respectively. The definitions of *t* and *t*_0_ depend on the relationship between *i* and *j*: (1) for twin pairs, *t* = chronological age and *t*_0_ = 18 years; (2) for sibling pairs, *t* = chronological age of the younger sibling and *t*_0_ = chronological age of the younger sibling when the older sibling was 18 years old; (3) for parent–offspring pairs, *t* = chronological age of the offspring and *t*_0_ = 18 years; and (4) for spouse pairs, *t* = time in years since the pair married (assumed to be the average chronological age of the pair minus 24 years) and *t*_0_ = time in years when the pair became separated (if known).

For individuals *i* and *j* from the same family, their covariance was modeled as

$${\text{COV}}_{ij}=\left\{\begin{array}{ll}\alpha {\sigma }_{A}^{2}+{\beta }_{A}\left(1- {\text{e}}^{-{\lambda }_{A}t}\right)+{\beta }_{C}(1- {\text{e}}^{-{\lambda }_{C}t})&\quad \mathrm{if }\quad t\le {t}_{0}\\ \alpha {\sigma }_{A}^{2}+{\beta }_{A}\left(1- {\text{e}}^{-{\lambda }_{A}{t}_{0}}\right){\text{e}}^{-{\upnu }_{A}\left(t-{t}_{0}\right)}+{\beta }_{C}(1- {\text{e}}^{-{\lambda }_{C}{t}_{0}}){\text{e}}^{-{\nu }_{C}(t-{t}_{0})},&\quad \mathrm{if}\quad t>{t}_{0}\end{array}\right.$$

where *α*, *β*_A_, *β*_C_, *λ*_A_, *λ*_C_, *υ*_A_, *υ*_C_ ≥ 0, and the definitions of *t* and *t*_0_ are the same as above.

We assumed that the variation of DNAm age can be caused by combinations of additive genetic factors (A), shared environmental factors (C), and individual-specific environmental factors (E). We assessed model fits using the Akaike information criterion (AIC) for the following models and assumptions:

1. AE model: variation is caused by only A and E; the effects of A are constant across the lifespan; *α* = 2 × kinship coefficient, *β*_A_, *β*_C_, *λ*_A_, *λ*_C_, *υ*_A_, *υ*_C_ = 0, and *σ*_A_^2^ is free to be estimated.
2. Cohabitation-dependent AE model: variation is caused only by A and E; the effects of A depend on cohabitation; *α*, *σ*_A_^2^, *β*_C_, *λ*_C_, *υ*_C _= 0, *β*_A _= 2 × kinship coefficient, *λ*_A_, *υ*_A _= 0 for spouse pairs, and the same and free to be estimated for the other pairs.
3. Cohabitation-dependent ACE model: variation is caused by A, C and E; the effects of A and C both depend on cohabitation; *α*, *σ*_A_^2^ = 0, *β*_A_ = 2 × kinship coefficient, *λ*_A_, *υ*_A _= 0 for spouse pairs, but the same and free to be estimated for the other pairs, *β*_C_ = 1 for all pairs, *λ*_C_ and *υ*_C_ are the same for MZ, DZ, sibling and parent–offspring pairs and free to be estimated.
4. Cohabitation-dependent CE model: variation is caused by only C and E; the effects of C depend on cohabitation; *α*, *σ*_A_^2^, *β*_A_, *λ*_A_, *υ*_A_ = 0, *β*_C_ = 1 for DZ, sibling pairs and spouse pairs, and free to be estimated for the other pairs, *λ*_C_ and *υ*_C_ are the same for MZ, DZ, sibling and parent–offspring pairs and free to be estimated.

Under the above cohabitation-dependent CE model, we further allowed for the role of additive genetic factors whose effects were assumed to be constant across the lifespan. This was made possible by letting *α* = 2 × kinship coefficient and *σ*_A_^2^ ≠ 0. *σ*_A_^2^ was estimated.

## Supplementary information

**Additional file 1.** Supplementary Methods and Tables.

** Additional file 2: Figure S1.** Correlation between chronological age and skin-blood DNAm age within each study. PETS: Peri/postnatal Epigenetic Twins Study; BSGS: Brisbane System Genetics Study; E-Risk: Environmental Risk Longitudinal Twin Study; DTR: Danish Twin Registry; AMDTSS: Australian Mammographic Density Twins and Sisters Study; MuTHER: Multiple Tissue Human Expression Resource Study; OATS: Older Australian Twins Study; LSADT: Longitudinal Study of Aging Danish Twins; MCCS: Melbourne Collaborative Cohort Study.

** Additional file 3: Figure S2.** Correlation between chronological age and Han’s DNAm age within each study. PETS: Peri/postnatal Epigenetic Twins Study; BSGS: Brisbane System Genetics Study; E-Risk: Environmental Risk Longitudinal Twin Study; DTR: Danish Twin Registry; AMDTSS: Australian Mammographic Density Twins and Sisters Study; OATS: Older Australian Twins Study; LSADT: Longitudinal Study of Aging Danish Twins; MCCS: Melbourne Collaborative Cohort Study.

** Additional file 4: Figure S3.** Variance in age-adjusted skin-blood DNAm age by chronological age. PETS: Peri/postnatal Epigenetic Twins Study; BSGS: Brisbane System Genetics Study; E-Risk: Environmental Risk Longitudinal Twin Study; DTR: Danish Twin Registry; AMDTSS: Australian Mammographic Density Twins and Sisters Study; MuTHER: Multiple Tissue Human Expression Resource Study; OATS: Older Australian Twins Study; LSADT: Longitudinal Study of Aging Danish Twins; MCCS: Melbourne Collaborative Cohort Study.

** Additional file 5: Figure S4.** Variance in age-adjusted Han’s DNAm age by chronological age. PETS: Peri/postnatal Epigenetic Twins Study; BSGS: Brisbane System Genetics Study; E-Risk: Environmental Risk Longitudinal Twin Study; DTR: Danish Twin Registry; AMDTSS: Australian Mammographic Density Twins and Sisters Study; OATS: Older Australian Twins Study; LSADT: Longitudinal Study of Aging Danish Twins; MCCS: Melbourne Collaborative Cohort Study.

## Data Availability

The datasets (accession number) are available on Gene Expression Omnibus: PETS 27K (GSE36642), PETS 450K (GSE42700), BSGS (GSE56105), E-Risk (GSE105018), DTR (GSE61496), AMDTSS (GSE100227), TwinsUK (GSE58045), LSADT (GSE73115). The MuTHER dataset is available on ArrayExpress under the accession number of E-MTAB-1866.

## Abbreviations

DNAm: DNA methylation
SNP: Single nucleotide polymorphism
PETS: Peri/postnatal Epigenetic Twins Study
BSGS: Brisbane System Genetics Study
E-Risk: Environmental Risk Longitudinal Twin Study
DTR: Danish Twin Registry
AMDTSS: Australian Mammographic Density Twins and Sisters Study
MuTHER: Multiple Tissue Human Expression Resource Study
OATS: Older Australian Twins Study
LSADT: Longitudinal Study of Aging Danish Twins Study
MCCS: Melbourne Collaborative Cohort Study
MZ: Monozygotic twin
DZ: Dizygotic twin
A: Additive genetic factors
C: Shared environmental factors
E: Individual-specific environmental factors
AIC: Akaike information criterion
