# Supplementary material for: Genetic and environmental causes of variation in epigenetic aging across the lifespan
Source: Clin Epigenetics. 2020 Oct 22;12:158. doi: 10.1186/s13148-020-00950-1 (PMC7583207; doi:10.1186/s13148-020-00950-1)
Supplement: Supplementary file 1 — Additional file 1. Supplementary Methods and Tables. [file 13148_2020_950_MOESM1_ESM.docx]

**Table of Contents**

[Study sample 2](#_Toc50465068)

[References 7](#_Toc50465069)

[Table S1 Within-study familial correlation estimates in DNAm age additionally adjusting for blood cell composition 10](#_Toc50465070)

[Table S2 Results from modelling the familial correlations in DNAm age across the lifespan as a function of cohabitation history 11](#_Toc50465071)

[Table S3 Results from modelling the causes of variation in DNAm age across the lifespan 12](#_Toc50465072)

[Table S4 Characteristics of the skin-blood clock and Han’s clock by study 13](#_Toc50465073)

[Table S5 Within-study familial correlation estimates in the skin-blood clock and Han’s clock 14](#_Toc50465074)

# **Study sample**

1. Peri/postnatal Epigenetic Twins Study (PETS)

The PETS is an Australian twin birth cohort study of the plasticity of epigenetic marks during the intrauterine period and early childhood[1]. A total of 250 newborn twin pairs were recruited between 2007 and 2009, and data on maternal factors during pregnancy, infant anthropometric measurements, and biological specimens from different cell lineages were collected at several time points. The study was approved by the Human Research Ethics Committees of the Royal Women’s Hospital, Mercy Hospital for Women, and Monash Medical Centre, Melbourne. The PETS included three datasets measured using the HumanMethylation27 array (27K), the HumanMethylation450 array (450K), and the HumanMethylationEPIC array (EPIC).

The 27K dataset was accessed from the Gene Expression Omnibus (GEO) with the accession number GSE36642 for 22 newborn monozygotic twin (MZ) pairs and 11 dizygotic twin (DZ) pairs. DNA was extracted from cord blood mononuclear cells (18 MZ, 9 DZ pairs), human umbilical vascular endothelial cells (14 MZ, 10 DZ pairs) and placenta (8 MZ, 7 DZ pairs). Data were background corrected, normalized and minimized for batch effects using *lumi* package; see Gordon *et al*. for more details[2].

The 450K dataset was accessed from the GEO with the accession number GSE42700. This dataset included longitudinal measures for 10 MZ pairs and 5 DZ pairs: DNA was extracted from buccal cells collected at birth and age 18 months, respectively. Data were background corrected, pre-processed using the Illumina method within the Bioconductor *minfi* package[3] and normalized using the subset-quantile within-array normalization (SWAN) method[4]; see Martino *et al.* for more details[5].

The EPIC dataset included 23 newborn MZ pairs and 22 DZ pairs. DNA was extracted from cord blood. Data were background corrected, pre-processed using the *minfi* package[3] and normalized using the quantile normalization method.

2. Brisbane Systems Genetic Study (BSGS)

The BSGS is an Australian twin family study of pigmented nevi and cognition comprising adolescent twins, their siblings and parents[6]. This analysis included 614 participants of European descent. The study was approved by the Human Research Ethics Committee of the Queensland Institute for Medical Research.

The DNA methylation dataset was accessed from the GEO with the accession number GSE56105. DNA was extracted from peripheral blood and methylation was measured using the Infinium HumanMethylation450 BeadChip. Individual probes were normalized across all samples using a generalized linear model with a logistic link function; see McRae *et al.* for more details[7].

3. E-Risk Longitudinal Twin Study (E-Risk)

The E-Risk tracks the development of a 1994–1995 birth cohort of 2,232 British children. It comprises 56% MZ and 44% DZ pairs. This analysis included 426 MZ pairs and 306 DZ pairs aged 18 years. The study was approved by the NRES Committee London — Camberwell St Giles Ethics Committee, and the Joint South London and Maudsley and the Institute of Psychiatry Research Ethics Committee approved each phase of the E-Risk study.

The DNA methylation dataset was accessed from the GEO with the accession number GSE105018. DNA was extracted from blood samples and methylation was measured using the Infinium HumanMethylation450 BeadChip. Data were normalized with the *dasen* function from the *wateRmelon* package; see Hannon *et al.* for more details[8].

4. Danish Twin Registry (DTR)

MZ pairs discordant for birth weight were sampled based on information from the Danish Twin Registry. This analysis included 150 MZ pairs in two age groups, younger adults (mean age 33 years) and older adults (mean age 63 years). The study was approved by The Regional Scientific Ethical Committees for Southern Denmark.

The DNA methylation dataset was accessed from the GEO with the accession number GSE61496. DNA was extracted from blood samples and methylation was measured using the Infinium HumanMethylation450 BeadChip. Data were normalized using the SWAN method[4] within the *minfi* package[3]; see Tan *et al.* for more details[9].

5. Australian Mammographic Density Twins and Sisters Study (AMDTSS)

The AMDTSS is an Australian twin family study principally of mammographic density[10]. Participants completed questionnaire surveys through telephone-administered interviews and donated blood samples. This analysis included 479 middle-aged women selected for DNA methylation research. The study was approved by the Australian Twin Registry and the Human Research Ethics Committee of the University of Melbourne.

The DNA methylation dataset was available on the GEO under the accession number of GSE100227. DNA was extracted from dried blood spots stored on Guthrie cards and DNA methylation was measured using the Infinium HumanMethylation450 BeadChip. Data was processed by Bioconductor *minfi* package[3], which included normalization of data using Illumina’s reference factor-based normalization methods (*preprocessIllumina*) and the SWAN method[4] for type I and II probe bias correction. An empirical Bayes batch-effects removal method *ComBat*[11] was applied to minimize the technical variation across batches. See Li *et al.* for more details[12].

6. TwinsUK cohort

The TwinsUK cohort comprises unselected volunteers ascertained from the general population. Means and ranges of quantitative phenotypes in Twins UK were similar to age-matched samples from the general population in the UK. This analysis included 33 middle-aged female MZ pairs and 43 middle-aged female DZ pairs.

The DNA methylation dataset was accessed from the GEO under the accession number GSE58045. DNA was extracted from blood samples and methylation was measured using the Infinium HumanMethylation27 BeadChip; see Bell *et al.* for more details [13].

7. Multiple Tissue Human Expression Resource (MuTHER) Study

The MuTHER is a study of middle-aged females, including 386 twin pairs and 84 singletons of European descent recruited through the TwinsUK Adult Twin Registry[14]. This analysis included 246 twin pairs. The study was approved by the Research Ethics Committee of St. Thomas’ Hospital, London. All participants provided written informed consent.

The DNA methylation dataset was accessed from the ArrayExpress with the accession number E-MTAB-1866. DNA was extracted from adipose tissue samples and methylation was measured using the Infinium HumanMethylation450 BeadChip. Data were quantile normalized; see Grundberg *et al.* for more details[14].

8. Older Australian Twins Study (OATS)

The OATS is an Australian longitudinal, multi-center study of twins aged 65 years and older that commenced in 2007 investigating healthy brain aging[15]. This analysis included 108 MZ pairs. The study was approved by the Australian Twin Registry and the ethics committees of the University of New South Wales, University of Melbourne, Queensland Institute of Medical Research and the South Eastern Sydney and Illawarra Area Health Service. All participants provided written informed consent.

DNA was extracted from blood samples and methylation was measured using the Infinium HumanMethylation450 BeadChip. Raw intensity data were background corrected and methylation beta-values were generated using the R *minfi* package[3]. The SWAN method[4] was performed for type I and II probe bias correction. Probes not detected in all samples were removed, as were probes containing SNPs and probes on the sex chromosomes.

9. Longitudinal Study of Aging Danish Twins (LSADT)

The LSADT collected same-sex twin pairs born in Denmark for longitudinal assessment for aging-related phenotypes. This analysis included 43 elderly twin pairs.

The DNA methylation dataset was assessed from the GEO under the accession number GSE73115. The dataset included longitudinal measurements: DNA was extracted from blood samples collected at years 1997 and 2007, respectively. Methylation was measured using the Infinium HumanMethylation450 BeadChip. Data were normalized using the SWAN method[4] within the *minfi* package[3]; see Tan *et al.* for more details[16].

10. Melbourne Collaborative Cohort Study (MCCS)

The MCCS is an Australian prospective cohort study of 41,514 healthy adult volunteers (24,469 women, 17,045 men) aged between 27 and 76 years (99.3% aged 40−69 years) recruited between 1990 and 1994[17]. Peripheral blood samples were obtained from participants at baseline and 5,629 participants from six nested cancer case-control studies were measured for DNA methylation. This analysis included 62 spouse pairs from controls. The study was approved by the Cancer Council Victoria’s Human Research Ethics Committee and performed in accordance with the institution’s ethical guidelines. All participants provided written informed consent.

Samples in each case-control sub-study were processed separately during non-overlapping periods of time over a two-year period in the same laboratory with the same protocol. DNA was extracted from peripheral blood samples collected at baseline, prior to any diagnosis of cancer. Methylation was measured using the Infinium HumanMethylation450 BeadChip. The same data pre-processing procedure was applied to each case-control sub-study, respectively. Raw intensity data was processed by Bioconductor *minfi* package[3], which included normalzation of data using Illumina’s reference factor-based normalization methods (*preprocessIllumina*) and the SWAN method[4] for type I and II probe bias correction. *ComBat*[11] was applied to the data from all samples across sub-studies to minimize the influence of chip effects; see Severi *et al*.[18] and Wong *et al.*[19] for more details.

# **References**

1. Saffery R, Morley R, Carlin JB, Joo JH, Ollikainen M, Novakovic B, et al: Cohort profile: The peri/post-natal epigenetic twins study. *Int J Epidemiol* 2012, 41:55-61.

2. Gordon L, Joo JE, Powell JE, Ollikainen M, Novakovic B, Li X, et al: Neonatal DNA methylation profile in human twins is specified by a complex interplay between intrauterine environmental and genetic factors, subject to tissue-specific influence. *Genome Res* 2012, 22:1395-1406.

3. Aryee MJ, Jaffe AE, Corrada-Bravo H, Ladd-Acosta C, Feinberg AP, Hansen KD, et al: Minfi: a flexible and comprehensive Bioconductor package for the analysis of Infinium DNA methylation microarrays. *Bioinformatics* 2014, 30:1363-1369.

4. Maksimovic J, Gordon L, Oshlack A: SWAN: Subset-quantile within array normalization for illumina infinium HumanMethylation450 BeadChips. *Genome Biol* 2012, 13:R44.

5. Martino D, Loke YJ, Gordon L, Ollikainen M, Cruickshank MN, Saffery R, et al: Longitudinal, genome-scale analysis of DNA methylation in twins from birth to 18 months of age reveals rapid epigenetic change in early life and pair-specific effects of discordance. *Genome Biol* 2013, 14:R42.

6. Powell JE, Henders AK, McRae AF, Caracella A, Smith S, Wright MJ, et al: The Brisbane Systems Genetics Study: genetical genomics meets complex trait genetics. *PLoS One* 2012, 7:e35430.

7. McRae AF, Powell JE, Henders AK, Bowdler L, Hemani G, Shah S, et al: Contribution of genetic variation to transgenerational inheritance of DNA methylation. *Genome Biol* 2014, 15:R73.

8. Hannon E, Knox O, Sugden K, Burrage J, Wong CCY, Belsky DW, et al: Characterizing genetic and environmental influences on variable DNA methylation using monozygotic and dizygotic twins. *PLoS Genet* 2018, 14:e1007544.

9. Tan Q, Frost M, Heijmans BT, von Bornemann Hjelmborg J, Tobi EW, Christensen K, et al: Epigenetic signature of birth weight discordance in adult twins. *BMC Genomics* 2014, 15:1062.

10. Odefrey F, Stone J, Gurrin LC, Byrnes GB, Apicella C, Dite GS, et al: Common genetic variants associated with breast cancer and mammographic density measures that predict disease. *Cancer Res* 2010, 70:1449-1458.

11. Johnson WE, Li C, Rabinovic A: Adjusting batch effects in microarray expression data using empirical Bayes methods. *Biostatistics* 2007, 8:118-127.

12. Li S, Wong EM, Joo JE, Jung CH, Chung J, Apicella C, et al: Genetic and Environmental Causes of Variation in the Difference Between Biological Age Based on DNA Methylation and Chronological Age for Middle-Aged Women. *Twin Res Hum Genet* 2015, 18:720-726.

13. Bell JT, Tsai PC, Yang TP, Pidsley R, Nisbet J, Glass D, et al: Epigenome-wide scans identify differentially methylated regions for age and age-related phenotypes in a healthy ageing population. *PLoS Genet* 2012, 8:e1002629.

14. Grundberg E, Meduri E, Sandling JK, Hedman AK, Keildson S, Buil A, et al: Global analysis of DNA methylation variation in adipose tissue from twins reveals links to disease-associated variants in distal regulatory elements. *Am J Hum Genet* 2013, 93:876-890.

15. Sachdev PS, Lammel A, Trollor JN, Lee T, Wright MJ, Ames D, et al: A comprehensive neuropsychiatric study of elderly twins: the Older Australian Twins Study. *Twin Res Hum Genet* 2009, 12:573-582.

16. Tan Q, Heijmans BT, Hjelmborg JV, Soerensen M, Christensen K, Christiansen L: Epigenetic drift in the aging genome: a ten-year follow-up in an elderly twin cohort. *Int J Epidemiol* 2016.

17. Giles GG, English DR: The Melbourne Collaborative Cohort Study. *IARC Sci Publ* 2002, 156:69-70.

18. Severi G, Southey MC, English DR, Jung CH, Lonie A, McLean C, et al: Epigenome-wide methylation in DNA from peripheral blood as a marker of risk for breast cancer. *Breast Cancer Res Treat* 2014, 148:665-673.

19. Wong Doo N, Makalic E, Joo JE, Vajdic CM, Schmidt DF, Wong EM, et al: Global measures of peripheral blood-derived DNA methylation as a risk factor in the development of mature B-cell neoplasms. *Epigenomics* 2016, 8:55-66.

# **Table S1 Within-study familial correlation estimates in DNAm age additionally adjusting for blood cell composition**

| Study* | Type of pairs | Number of pairs | Correlation (95% CI) | P | P for MZ vs DZ |
| --- | --- | --- | --- | --- | --- |
| PETS EPIC | MZ | 23 | 0.22 (-0.14 to 0.53) | 0.23 | 0.39 |
|  | DZ | 22 | -0.04 (-0.46 to 0.10) | 0.87 |  |
|  | MZ and DZ | 45 | 0.11 (-0.14 to 0.38) | 0.44 |  |
| BSGS | MZ | 67 | 0.68 (0.61 to 0.73) | <0.001 | <0.001 |
|  | DZ | 111 | 0.31 (0.15 to 0.45) | <0.001 |  |
|  | Siblings | 260 | 0.29 (0.17 to 0.40) | <0.001 |  |
|  | Parent-offspring | 363 | 0.12 (0.00 to 0.25) | 0.06 |  |
|  | Spouses | 59 | -0.06 (-0.29 to 0.18) | 0.63 |  |
| E-Risk | MZ | 426 | 0.45 (0.39 to 0.51) | <0.001 | 0.23 |
|  | DZ | 306 | 0.38 (0.30 to 0.45) | <0.001 |  |
|  | MZ and DZ | 732 | 0.42 (0.37 to 0.47) | <0.001 |  |
| DTR younger adults | MZ | 73 | 0.54 (0.42 to 0.65) | <0.001 | − |
| AMDTSS | MZ | 66 | 0.43 (0.26 to 0.58) | <0.001 | 0.05 |
|  | DZ | 66 | 0.12 (-0.10 to 0.34) | 0.28 |  |
|  | MZ and DZ | 132 | 0.26 (0.10 to 0.40) | 0.002 |  |
|  | Siblings | 552 | 0.09 (0.00 to 0.18) | 0.06 |  |
| DTR older adults | MZ | 77 | 0.40 (0.23 to 0.54) | <0.001 | − |
| OATS | MZ | 108 | 0.36 (0.21 to 0.49) | <0.001 | − |
| LSADT 1997 | MZ | 18 | 0.00 (-0.55 to 0.55) | 1.00 | 0.36 |
|  | DZ | 25 | 0.31 (0.01 to 0.56) | 0.05 |  |
|  | MZ and DZ | 43 | 0.24 (-0.04 to 0.48) | 0.10 |  |
| LSADT 2007 | MZ | 18 | 0.38 (-0.01 to 0.67) | 0.07 | 0.72 |
|  | DZ | 25 | 0.28 (-0.05 to 0.55) | 0.10 |  |
|  | MZ and DZ | 43 | 0.32 (0.06 to 0.54) | 0.02 |  |
| MCCS | Spouses | 62 | 0.10 (-0.14 to 0.33) | 0.42 | − |

Abbreviations – MZ: monozygotic twin; DZ: dizygotic twin; CI: confidence interval

*Studies – PETS: Peri/postnatal Epigenetic Twins Study EPIC array dataset; BSGS: Brisbane System Genetics Study; E-Risk: Environmental Risk Longitudinal Twin Study; DTR: Danish Twin Registry, in two groups: younger and older adults; AMDTSS: Australian Mammographic Density Twins and Sisters Study; OATS: Older Australian Twins Study; LSADT: Longitudinal Study of Aging Danish Twins, with samples collected at years 1997 and 2007, respectively; MCCS: Melbourne Collaborative Cohort Study

# **Table S2 Results from modelling the familial correlations in DNAm age across the lifespan as a function of cohabitation history**

| Pairs | Parameter | All parameters freely estimated | |  | All θ=1 | |  | DZ=Sibling, ν is the same for all pairs | |
| --- | --- | --- | --- | --- | --- | --- | --- | --- | --- |
|  |  | Estimate (SE) | P |  | Estimate (SE) | P |  | Estimate (SE) | P |
| MZ | θ | 1.20 (0.13) | 0.11* |  | 1 |  |  | 1 |  |
|  | λ | 0.021 (0.011) | 0.05 |  | 0.040 (0.003) | <0.001 |  | 0.041 (0.003) | <0.001 |
|  | ν | 0.003 (0.002) | 0.11 |  | 0.003 (0.002) | 0.10 |  | 0.004 (0.002) | 0.02 |
| DZ | θ | 1.03 (0.15) | 0.87* |  | 1 |  |  | 1 |  |
|  | λ | 0.026 (0.014) | 0.07 |  | 0.028 (0.004) | <0.001 |  | 0.026 (0.003) | <0.001 |
|  | ν | 0.004 (0.004) | 0.28 |  | 0.004 (0.004) | 0.28 |  | 0.004 (0.002) | 0.02 |
| Sibling | θ | 1.09 (0.34) | 0.80* |  | 1 |  |  | 1 |  |
|  | λ | 0.018 (0.032) | 0.58 |  | 0.026 (0.006) | <0.001 |  | 0.026 (0.003) | <0.001 |
|  | ν | 0.019 (0.011) | 0.08 |  | 0.018 (0.010) | 0.08 |  | 0.004 (0.002) | 0.02 |
| Parent-offspring | θ | 0.69 (0.54) | 0.56* |  | 1 |  |  | 1 |  |
|  | λ | 0.043 (0.069) | 0.54 |  | 0.012 (0.005) | 0.02 |  | 0.011 (0.005) | 0.02 |
| Spouse | θ | 0.76 (0.44) | 0.58* |  | 1 |  |  | 1 |  |
|  | λ | 0.012 (0.022) | 0.58 |  | 0.003 (0.003) | 0.44 |  | 0.003 (0.003) | 0.44 |
| Log-likelihood |  | -1907.195 | |  | -1908.715 | |  | -1911.432 | |

Abbreviations – MZ: monozygotic twin; DZ: dizygotic twin; SE: standard error

*P-value from comparing the estimate with 1

# **Table S3 Results from modelling the causes of variation in DNAm age across the lifespan**

| Parameter | Pairs | AE model | Cohabitation-dependent AE model | Cohabitation-dependent ACE model | Cohabitation-dependent CE model |
| --- | --- | --- | --- | --- | --- |
| α | MZ | 1 | 0 | 0 | 0 |
|  | DZ and sibling | 0.5 | 0 | 0 | 0 |
|  | Parent-offspring | 0.5 | 0 | 0 | 0 |
|  | Spouse | 0 | 0 | 0 | 0 |
| σ_A_^2^ (SE) | All pairs | 0.52 (0.02) | 0 | 0 | 0 |
| β_A_ | MZ | 0 | 1 | 1 | 0 |
|  | DZ and sibling | 0 | 0.5 | 0.5 | 0 |
|  | Parent-offspring | 0 | 0.5 | 0.5 | 0 |
|  | Spouse | 0 | 0 | 0 | 0 |
| λ_A_ (SE) | MZ | 0 | 0.041 (0.003) | 0.025 (0.007) | 0 |
|  | DZ and sibling | 0 | 0.041 (0.003) | 0.025 (0.007) | 0 |
|  | Parent-offspring | 0 | 0.041 (0.003) | 0.025 (0.007) | 0 |
|  | Spouse | 0 | 0 | 0 | 0 |
| ν_A_ (SE) | All pairs | 0 | 0.003 (0.002) | 0.003 (0.006) | 0 |
| β_C_ (SE) | MZ | 0 | 0 | 1 | 1.41 (0.13) |
|  | DZ and sibling | 0 | 0 | 1 | 1 |
|  | Parent-offspring | 0 | 0 | 1 | 0.49 (0.20) |
|  | Spouse | 0 | 0 | 1 | 1 |
| λ_C_ (SE) | MZ | 0 | 0 | 0.009 (0.004) | 0.026 (0.003) |
|  | DZ and sibling | 0 | 0 | 0.009 (0.004) | 0.026 (0.003) |
|  | Parent-offspring | 0 | 0 | 0.009 (0.004) | 0.026 (0.003) |
|  | Spouse | 0 | 0 | 0.006 (0.014) | 0.003 (0.003) |
| ν_C_ (SE) | All pairs | 0 | 0 | 0.005 (0.003) | 0.004 (0.002) |
| Log-likelihood |  | -1943.141 | -1918.798 | -1915.739 | -1911.773 |
| AIC |  | 3892.282 | 3845.596 | 3845.478 | 3837.546 |

Abbreviations – MZ: monozygotic twin; DZ: dizygotic twin; SE: standard error; AIC: Akaike Information Criterion

Model details - AE model: variation was assumed to be caused by only additive genetic factors (A) and individual-specific environmental factors (E), and the effects of A are constant across the lifespan; Cohabitation-dependent AE model: variation was assumed to be caused by only A and E, and the effects of A depend on cohabitation; Cohabitation-dependent ACE model: variation was assumed to be caused by A, shared environmental factors (C) and E, and the effects of A and C both depend on cohabitation; Cohabitation-dependent CE model: variation is caused by only C and E, and the effects of C depend on cohabitation

#

# **Table S4 Characteristics of the skin-blood clock and Han’s clock by study**

| Study* | Type of pairs | Skin-blood clock | | |  | Han’s clock | | |
| --- | --- | --- | --- | --- | --- | --- | --- | --- |
|  |  | DNAm age, mean (SD) | Absolute deviation of DNAm and chronological ages, mean (SD) | Epigenetic age acceleration, mean (SD) |  | DNAm age, mean (SD) | Absolute deviation of DNAm and chronological ages, mean (SD) | Epigenetic age acceleration, mean (SD) |
| PETS EPIC | MZ | 0.7 (0.2) | 0.39 (0.20) | -0.03 (0.20) |  | -0.1 (0.16) | 0.16 (0.09) | -0.04 (0.16) |
|  | DZ | 0.8 (0.2) | 0.75 (0.16) | 0.03 (0.16) |  | -0.01 (0.16) | 0.12 (0.11) | 0.04 (0.16) |
| BSGS | MZ | 12.3 (2.5) | 1.75 (0.98) | -0.18 (1.29) |  | 12.0 (3.0) | 2.36 (1.50) | -0.02 (2.05) |
|  | DZ | 11.9 (2.5) | 1.60 (0.97) | 0.01 (1.27) |  | 11.4 (3.0) | 2.35 (1.48) | -0.01 (2.06) |
|  | Sibling | 14.4 (3.5) | 1.49 (0.97) | 0.01 (1.38) |  | 14.4 (4.2) | 2.19 (1.58) | 0.31 (2.40) |
|  | Spouse/parents | 47.6 (4.8) | 2.31 (1.72) | 0 (2.34) |  | 47.7 (5.8) | 3.03 (2.39) | 0 (3.57) |
| E-Risk | MZ | 26.5 (2.2) | 8.53 (2.20) | 0.01 (2.20) |  | 15.7 (2.8) | 3.01 (2.00) | 0.04 (2.82) |
|  | DZ | 26.5 (2.5) | 8.50 (2.49) | -0.01 (2.49) |  | 15.6 (2.9) | 3.15 (2.06) | -0.06 (2.93) |
| DTR younger adults | MZ | 36.3 (3.7) | 3.80 (2.34) | 0 (3.09) |  | 32.1 (3.8) | 2.86 (1.91) | 0 (3.32) |
| AMDTSS | MZ | 56.1 (6.7) | 3.66 (2.93) | -0.31 (3.77) |  | 52.1 (7.7) | 4.86 (3.93) | -0.35 (4.76) |
|  | DZ | 57.7 (5.6) | 4.19 (3.97) | 0.42 (4.43) |  | 53.8 (6.7) | 5.78 (5.16) | 0.47 (5.94) |
|  | Sibling | 56.9 (6.5) | 4.24 (3.29) | -0.07 (4.37) |  | 52.9 (7.0) | 5.46 (4.37) | -0.07 (5.09) |
| MuTHER | MZ | 54.4 (8.0) | 6.89 (4.34) | 0.10 (4.15) |  | − | − | − |
|  | DZ | 51.4 (8.6) | 6.37 (3.91) | -0.06 (4.14) |  | − | − | − |
| DTR older adults | MZ | 65.8 (5.3) | 3.32 (4.56) | 0 (3.72) |  | 61.4 (6.6) | 3.08 (3.32) | 0 (5.36) |
| OATS | MZ | 70.0 (5.5) | 4.01 (2.94) | 0 (3.91) |  | 67.3 (6.2) | 5.05 (3.64) | 0 (4.53) |
| LSADT 1997 | MZ | 75.2 (8.0) | 2.61 (1.88) | -0.77 (2.89) |  | 69.5 (5.7) | 8.11 (4.44) | -1.02 (5.79) |
|  | DZ | 76.1 (3.4) | 2.62 (1.99) | 0.22 (3.26) |  | 71.2 (5.5) | 6.21 (3.87) | 0.73 (5.45) |
| LSADT 2007 | MZ | 82.6 (3.3) | 4.18 (2.97) | -0.50 (3.44) |  | 77.2 (6.6) | 10.25 (5.38) | -0.45 (6.64) |
|  | DZ | 83.7 (3.4) | 3.65 (2.01) | 0.70 (3.38) |  | 78.0 (5.6) | 8.62 (4.42) | 0.32 (5.53) |
| MCCS | Spouse | 62.8 (6.4) | 4.21 (3.39) | 0 (4.41) |  | 58.4 (7.5) | 4.80 (3.46) | 0 (5.56) |

Abbreviations – MZ: monozygotic twin; DZ: dizygotic twin; SD: standard deviation

*Studies – PETS: Peri/postnatal Epigenetic Twins Study; BSGS: Brisbane System Genetics Study; E-Risk: Environmental Risk Longitudinal Twin Study; DTR: Danish Twin Registry, in two groups: younger and older adults; AMDTSS: Australian Mammographic Density Twins and Sisters Study; MuTHER: Multiple Tissue Human Expression Resource Study; OATS: Older Australian Twins Study; LSADT: Longitudinal Study of Aging Danish Twins, with samples collected at years 1997 and 2007, respectively; MCCS: Melbourne Collaborative Cohort Study

# **Table S5 Within-study familial correlation estimates in the skin-blood clock and Han’s clock**

| Study* | Type of pairs | Mean age | Skin-blood clock | | |  | Han’s clock | | |
| --- | --- | --- | --- | --- | --- | --- | --- | --- | --- |
|  |  |  | Correlation (95% CI) | P | P for MZ vs DZ |  | Correlation (95% CI) | P | P for MZ vs DZ |
| PETS EPIC | MZ | 0 | 0.40 (0.12 to 0.61) | 0.009 | 0.66 |  | 0.57 (0.38 to 0.72) | <0.001 | 0.10 |
|  | DZ | 0 | 0.50 (0.20 to 0.71) | 0.005 |  |  | 0.18 (-0.23 to 0.53) | 0.40 |  |
|  | MZ and DZ | 0 | 0.43 (0.22 to 0.60) | <0.001 |  |  | 0.38 (0.16 to 0.57) | 0.002 |  |
| BSGS | MZ | 13.8 | 0.66 (0.59 to 0.72) | <0.001 | <0.001 |  | 0.65 (0.57 to 0.72) | <0.001 | 0.001 |
|  | DZ | 13.2 | 0.25 (0.07 to 0.41) | 0.009 |  |  | 0.28 (0.12 to 0.43) | 0.001 |  |
|  | MZ and DZ | 13.5 | 0.41 (0.30 to 0.50) | <0.001 |  |  | 0.41 (0.30 to 0.51) | <0.001 |  |
|  | Siblings | 14.0 | 0.20 (0.07 to 0.33) | 0.005 |  |  | 0.31 (0.19 to 0.42) | <0.001 |  |
|  | Parent-offspring | 13.4 | 0.18 (0.07 to 0.33) | 0.002 |  |  | 0.24 (0.13 to 0.34) | <0.001 |  |
|  | Spouses | 46.6 | 0.15 (-0.08 to 0.37) | 0.20 |  |  | 0.13 (-0.12 to 0.35) | 0.31 |  |
| E-Risk | MZ | 18.0 | 0.58 (0.54 to 0.62) | <0.001 | <0.001 |  | 0.54 (0.49 to 0.58) | <0.001 | <0.001 |
|  | DZ | 18.0 | 0.33 (0.25 to 0.41) | <0.001 |  |  | 0.29 (0.20 to 0.37) | <0.001 |  |
|  | MZ and DZ | 18.0 | 0.46 (0.41 to 0.50) | <0.001 |  |  | 0.43 (0.38 to 0.47) | <0.001 |  |
| DTR younger adults | MZ | 33.1 | 0.42 (0.26 to 0.57) | <0.001 | − |  | 0.59 (0.48 to 0.68) | <0.001 | − |
| AMDTSS | MZ | 55.6 | 0.52 (0.38 to 0.64) | <0.001 | 0.12 |  | 0.55 (0.42 to 0.66) | <0.001 | 0.03 |
|  | DZ | 57.0 | 0.31 (0.13 to 0.47) | 0.001 |  |  | 0.26 (0.08 to 0.42) | 0.005 |  |
|  | MZ and DZ | 56.3 | 0.39 (0.26 to 0.50) | <0.001 |  |  | 0.36 (0.23 to 0.48) | <0.001 |  |
|  | Siblings | 56.4 | 0.25 (0.15 to 0.35) | <0.001 |  |  | 0.18 (0.07 to 0.28) | 0.002 |  |
| MuTHER | MZ | 58.4 | 0.50 (0.39 to 0.60) | <0.001 | 0.08 |  | − | − | − |
|  | DZ | 56.6 | 0.33 (0.20 to 0.45) | <0.001 |  |  | − | − |  |
|  | MZ and DZ | 57.3 | 0.40 (0.30 to 0.48) | <0.001 |  |  | − | − |  |
| DTR older adults | MZ | 63.2 | 0.50 (0.37 to 0.62) | <0.001 | − |  | 0.50 (0.37 to 0.62) | <0.001 | − |
| OATS | MZ | 71.2 | 0.52 (0.41 to 0.61) | <0.001 | − |  | 0.45 (0.33 to 0.56) | <0.001 | − |
| LSADT 1997 | MZ | 76.3 | 0.57 (0.32 to 0.74) | <0.001 | 0.03 |  | 0.37 (0.17 to 0.54) | 0.001 | 0.06 |
|  | DZ | 76.2 | -0.07 (-0.42 to 0.29) | 0.69 |  |  | 0.27 (-0.10 to 0.57) | 0.17 |  |
|  | MZ and DZ | 76.2 | 0.13 (-0.16 to 0.40) | 0.38 |  |  | 0.46 (0.25 to 0.62) | <0.001 |  |
| LSADT 2007 | MZ | 86.2 | 0.46 (0.16 to 0.69) | 0.008 | 0.10 |  | 0.36 (0.04 to 0.62) | 0.04 | 0.90 |
|  | DZ | 86.1 | -0.02 (-0.40 to 0.37) | 0.93 |  |  | 0.40 (0.06 to 0.65) | 0.03 |  |
|  | MZ and DZ | 86.1 | 0.19 (-0.10 to 0.44) | 0.21 |  |  | 0.38 (0.14 to 0.57) | 0.004 |  |
| MCCS | Spouses | 60.1 | 0.02 (-0.23 to 0.26) | 0.88 | − |  | 0.02 (-0.22 to 0.26) | 0.86 | − |

Abbreviations – MZ: monozygotic twin; DZ: dizygotic twin; SD: standard deviation

*Studies – PETS: Peri/postnatal Epigenetic Twins Study; BSGS: Brisbane System Genetics Study; E-Risk: Environmental Risk Longitudinal Twin Study; DTR: Danish Twin Registry, in two groups: younger and older adults; AMDTSS: Australian Mammographic Density Twins and Sisters Study; MuTHER: Multiple Tissue Human Expression Resource Study; OATS: Older Australian Twins Study; LSADT: Longitudinal Study of Aging Danish Twins, with samples collected at years 1997 and 2007, respectively; MCCS: Melbourne Collaborative Cohort Study
