# Supplementary figures and images for: Genetic and environmental causes of variation in epigenetic aging across the lifespan
Source: Clin Epigenetics. 2020 Oct 22;12:158. doi: 10.1186/s13148-020-00950-1 (PMC7583207; doi:10.1186/s13148-020-00950-1)

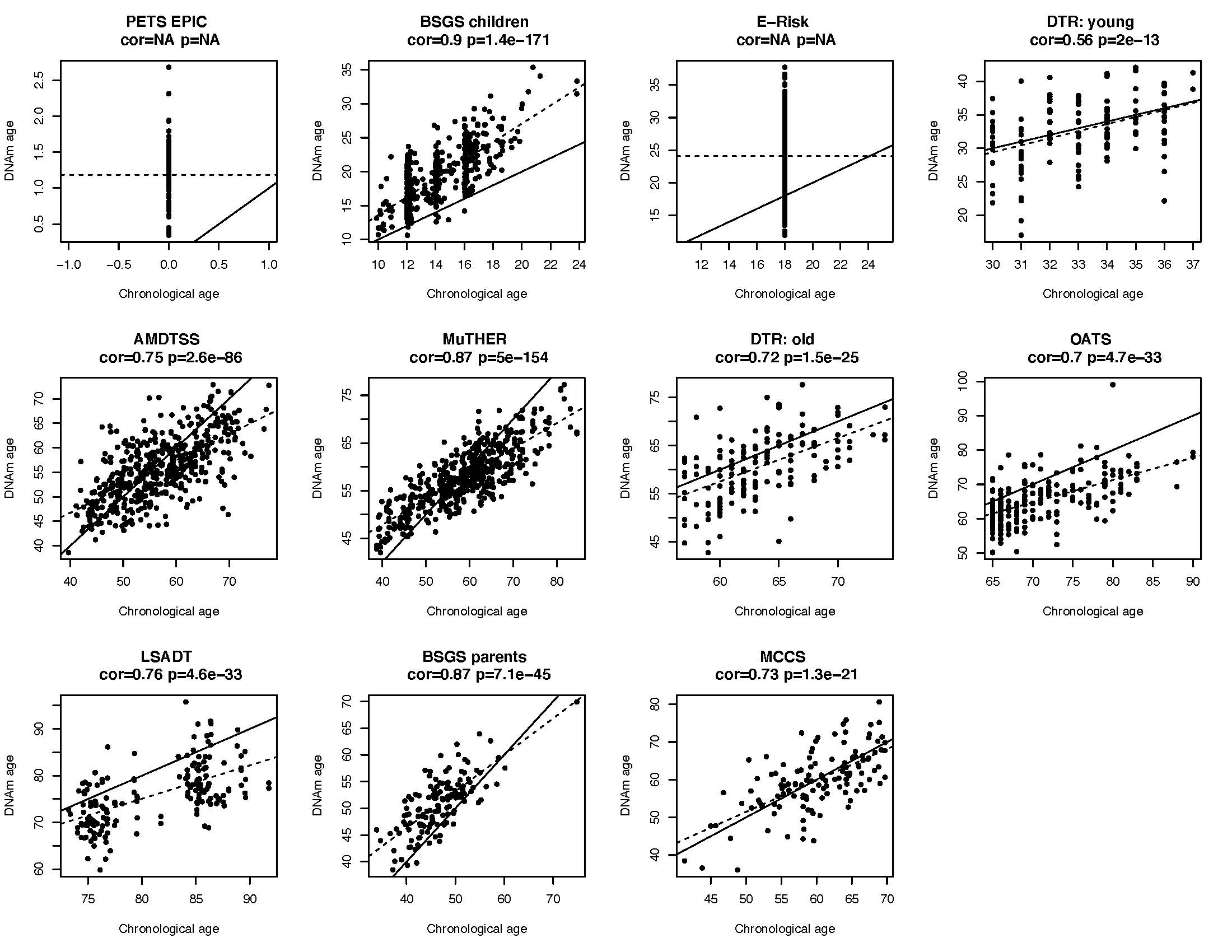

Supplement: Supplementary file 2 — Additional file 2: Figure S1. Correlation between chronological age and skin-blood DNAm age within each study. PETS: Peri/postnatal Epigenetic Twins Study; BSGS: Brisbane System Genetics Study; E-Risk: Environmental Risk Longitudinal Twin Study; DTR: Danish Twin Registry; AMDTSS: Australian Mammographic Density Twins and Sisters Study; MuTHER: Multiple Tissue Human Expression Resource Study; OATS: Older Australian Twins Study; LSADT: Longitudinal Study of Aging Danish Twins; MCCS: Melbourne Collaborative Cohort Study. [file 13148_2020_950_MOESM2_ESM.jpg]

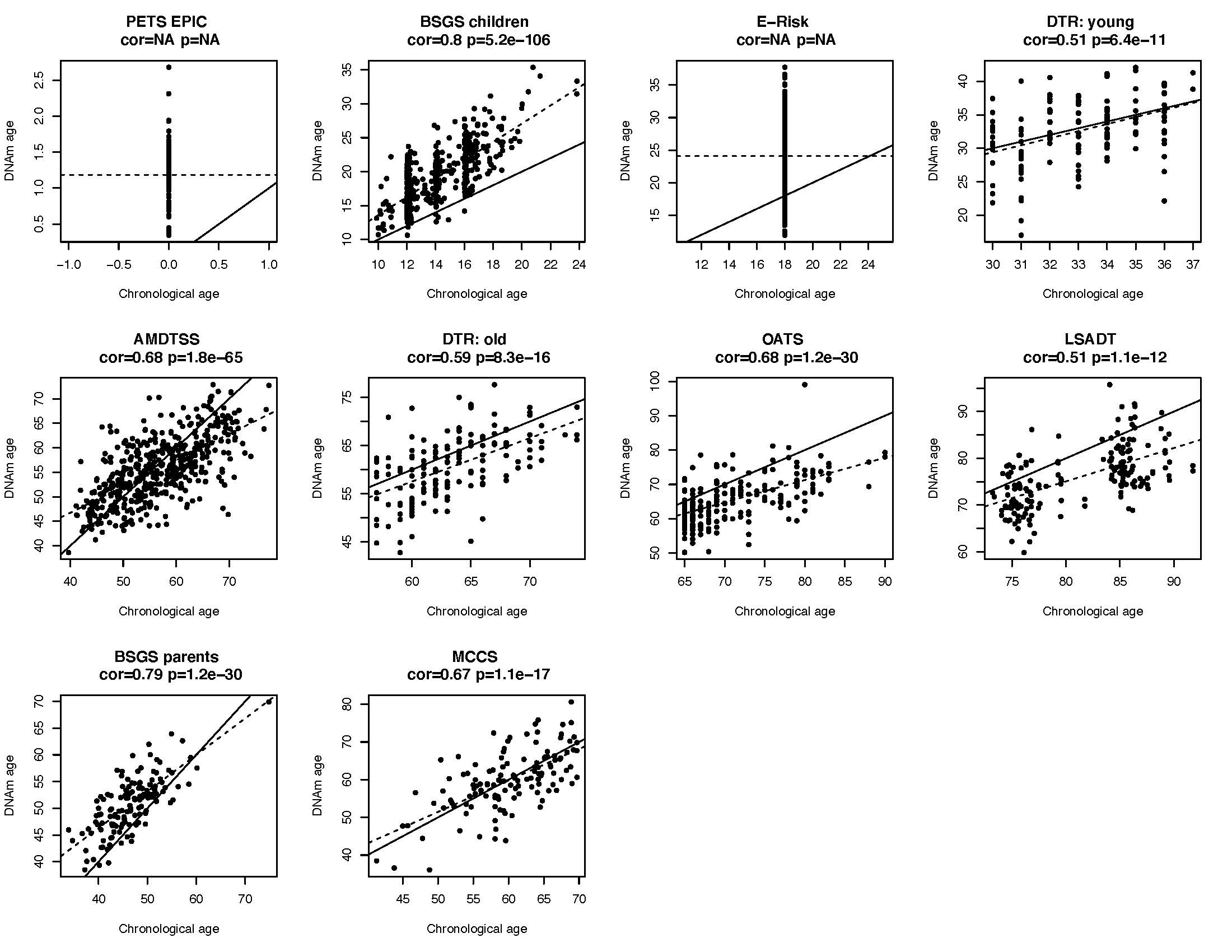

Supplement: Supplementary file 3 — Additional file 3: Figure S2. Correlation between chronological age and Han’s DNAm age within each study. PETS: Peri/postnatal Epigenetic Twins Study; BSGS: Brisbane System Genetics Study; E-Risk: Environmental Risk Longitudinal Twin Study; DTR: Danish Twin Registry; AMDTSS: Australian Mammographic Density Twins and Sisters Study; OATS: Older Australian Twins Study; LSADT: Longitudinal Study of Aging Danish Twins; MCCS: Melbourne Collaborative Cohort Study. [file 13148_2020_950_MOESM3_ESM.jpg]

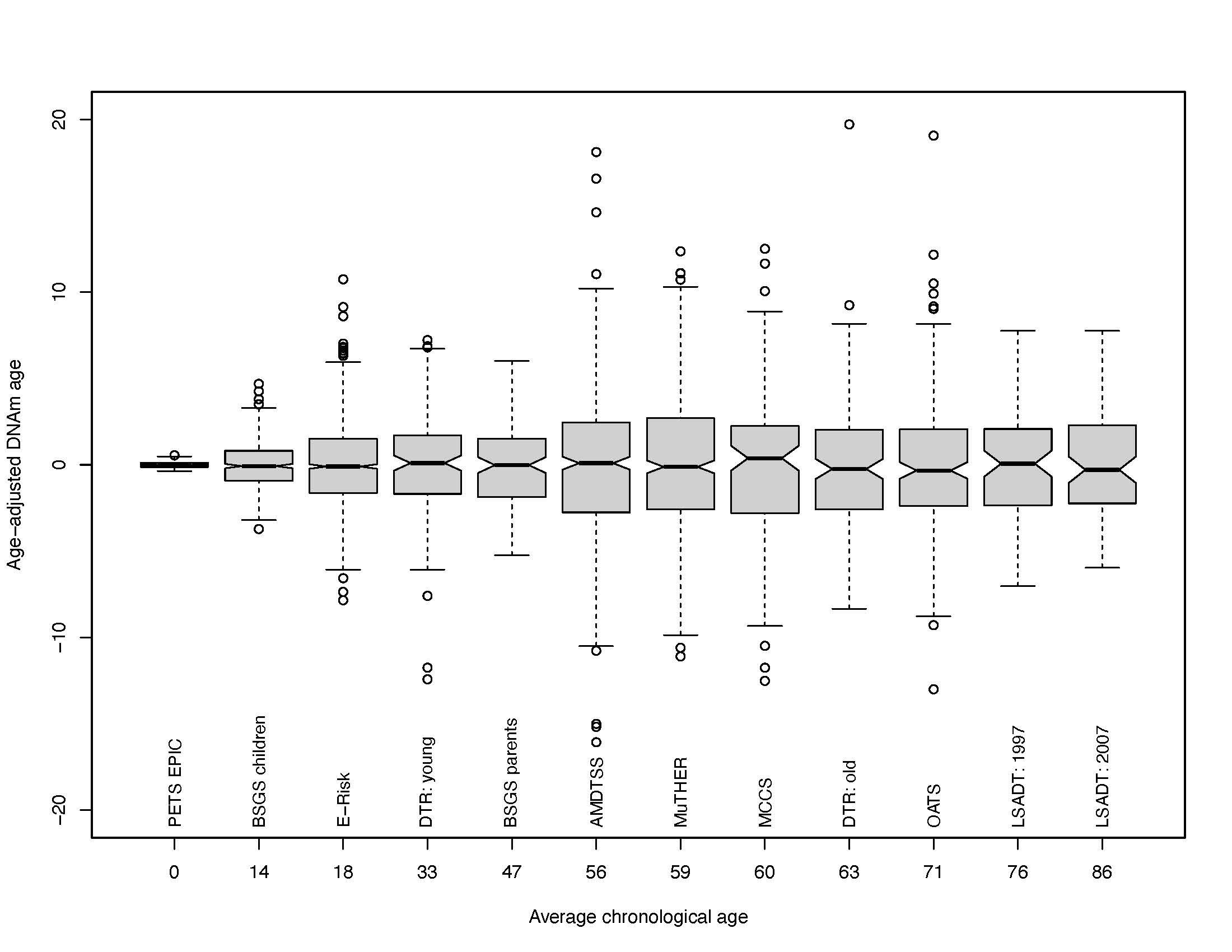

Supplement: Supplementary file 4 — Additional file 4: Figure S3. Variance in age-adjusted skin-blood DNAm age by chronological age. PETS: Peri/postnatal Epigenetic Twins Study; BSGS: Brisbane System Genetics Study; E-Risk: Environmental Risk Longitudinal Twin Study; DTR: Danish Twin Registry; AMDTSS: Australian Mammographic Density Twins and Sisters Study; MuTHER: Multiple Tissue Human Expression Resource Study; OATS: Older Australian Twins Study; LSADT: Longitudinal Study of Aging Danish Twins; MCCS: Melbourne Collaborative Cohort Study. [file 13148_2020_950_MOESM4_ESM.jpg]

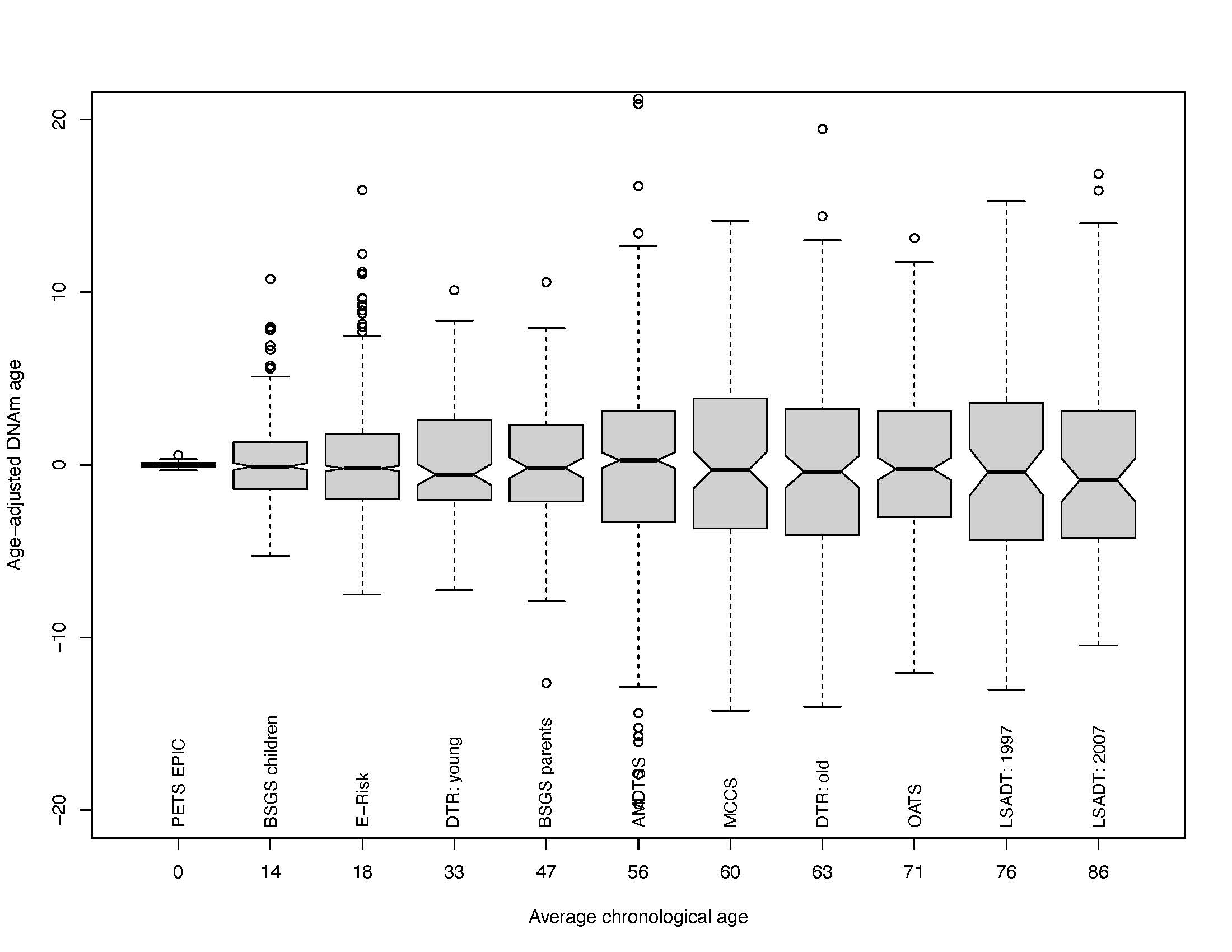

Supplement: Supplementary file 5 — Additional file 5: Figure S4. Variance in age-adjusted Han’s DNAm age by chronological age. PETS: Peri/postnatal Epigenetic Twins Study; BSGS: Brisbane System Genetics Study; E-Risk: Environmental Risk Longitudinal Twin Study; DTR: Danish Twin Registry; AMDTSS: Australian Mammographic Density Twins and Sisters Study; OATS: Older Australian Twins Study; LSADT: Longitudinal Study of Aging Danish Twins; MCCS: Melbourne Collaborative Cohort Study. [file 13148_2020_950_MOESM5_ESM.jpg]
